# Assessing the neuroprotective benefits for babies of antenatal magnesium sulphate: An individual participant data meta-analysis

**DOI:** 10.1371/journal.pmed.1002398

**Published:** 2017-10-04

**Authors:** Caroline A. Crowther, Philippa F. Middleton, Merryn Voysey, Lisa Askie, Lelia Duley, Peter G. Pryde, Stéphane Marret, Lex W. Doyle, for the AMICABLE Group

**Affiliations:** 1 Liggins Institute, University of Auckland, Auckland, New Zealand; 2 Australian Research Centre for Health of Women and Babies (ARCH), The Robinson Research Institute, Discipline of Obstetrics and Gynaecology, School of Medicine, The University of Adelaide, Adelaide, Australia; 3 Healthy Mothers Babies and Children, South Australian, Health and Medical Research Institute, Adelaide, Australia; 4 Nuffield Department of Primary Care Health Sciences, University of Oxford, Oxford, United Kingdom; 5 NHMRC Clinical Trials Centre, University of Sydney, Sydney, Australia; 6 Nottingham Clinical Trials Unit, Nottingham Health Science Partners, Queens Medical Centre, Nottingham, United Kingdom; 7 The University of Wisconsin Medical School, Madison, Wisconsin, United States of America; 8 Department of Neonatal Medicine and Neuropediatrics, Rouen University Hospital, Rouen, France; 9 INSERM U 1245, Neovasc team, Perinatal neurological handicap and Neuroprotection IRIB, School of Medicine, Normandy University, Rouen, France; 10 Department of Obstetrics and Gynaecology, The Royal Women’s’ Hospital, University of Melbourne, Australia; 11 Clinical Sciences, Murdoch Children’s Research Institute, Parkville, Victoria, Australia; 12 Department of Paediatrics, University of Melbourne, Parkville, Victoria, Australia; University of Manchester, UNITED KINGDOM

## Abstract

**Background:**

Babies born preterm are at an increased risk of dying in the first weeks of life, and those who survive have a higher rate of cerebral palsy (CP) compared with babies born at term. The aim of this individual participant data (IPD) meta-analysis (MA) was to assess the effects of antenatal magnesium sulphate, compared with no magnesium treatment, given to women at risk of preterm birth on important maternal and fetal outcomes, including survival free of CP, and whether effects differed by participant or treatment characteristics such as the reason the woman was at risk of preterm birth, why treatment was given, the gestational age at which magnesium sulphate treatment was received, or the dose and timing of the administration of magnesium sulphate.

**Methods and findings:**

Trials in which women considered at risk of preterm birth (<37 weeks’ gestation) were randomised to magnesium sulphate or control treatment and where neurologic outcomes for the baby were reported were eligible for inclusion. The primary outcomes were infant death or CP and severe maternal outcome potentially related to treatment. Studies were identified based on the Cochrane Pregnancy and Childbirth search strategy using the terms [antenatal or prenatal] and [magnesium] and [preterm or premature or neuroprotection or 'cerebral palsy']. The date of the last search was 28 February 2017. IPD were sought from investigators with eligible trials. Risk of bias was assessed using criteria from the Cochrane Collaboration. For each prespecified outcome, IPD were analysed using a 1-stage approach. All 5 trials identified were included, with 5,493 women and 6,131 babies. Overall, there was no clear effect of magnesium sulphate treatment compared with no treatment on the primary infant composite outcome of death or CP (relative risk [RR] 0.94, 95% confidence interval (CI) 0.85 to 1.05, 6,131 babies, 5 trials, *p* = 0.07 for heterogeneity of treatment effect across trials). In the prespecified sensitivity analysis restricted to data from the 4 trials in which the intent of treatment was fetal neuroprotection, there was a significant reduction in the risk of death or CP with magnesium sulphate treatment compared with no treatment (RR 0.86, 95% CI 0.75 to 0.99, 4,448 babies, 4 trials), with no significant heterogeneity (*p* = 0.28). The number needed to treat (NNT) to benefit was 41 women/babies to prevent 1 baby from either dying or having CP. For the primary outcome of severe maternal outcome potentially related to magnesium sulphate treatment, no events were recorded from the 2 trials providing data. When the individual components of the composite infant outcome were assessed, no effect was seen for death overall (RR 1.03, 95% CI 0.91 to 1.17, 6,131 babies, 5 trials) or in the analysis of death using only data from trials with the intent of fetal neuroprotection (RR 0.95, 95% CI 0.80 to 1.13, 4,448 babies, 4 trials). For cerebral palsy in survivors, magnesium sulphate treatment had a strong protective effect in both the overall analysis (RR 0.68, 95% CI 0.54 to 0.87, 4,601 babies, 5 trials, NNT to benefit 46) and the neuroprotective intent analysis (RR 0.68, 95% CI 0.53 to 0.87, 3,988 babies, 4 trials, NNT to benefit 42). No statistically significant differences were seen for any of the other secondary outcomes. The treatment effect varied little by the reason the woman was at risk of preterm birth, the gestational age at which magnesium sulphate treatment was given, the total dose received, or whether maintenance therapy was used. A limitation of the study was that not all trials could provide the data required for the planned analyses so that combined with low event rates for some important clinical events, the power to find a difference was limited.

**Conclusions:**

Antenatal magnesium sulphate given prior to preterm birth for fetal neuroprotection prevents CP and reduces the combined risk of fetal/infant death or CP. Benefit is seen regardless of the reason for preterm birth, with similar effects across a range of preterm gestational ages and different treatment regimens. Widespread adoption worldwide of this relatively inexpensive, easy-to-administer treatment would lead to important global health benefits for infants born preterm.

## Abstract 2

In an individual participant data meta-analysis, Caroline Crowther and colleagues examine the evidence for the neuroprotective benefits of antenatal magnesium sulphate for babies.

## Author summary

**Why was the study done?:**

- Effective therapies that can reduce the risk of neurological impairments and disabilities from preterm birth are needed.
- Antenatal magnesium sulphate given to women at imminent risk of preterm birth reduces the risk of cerebral palsy in their babies in aggregate data meta-analysis.
- The aims of the AMICABLE (Antenatal magnesium sulphate individual participant data international collaboration: Assessing the benefits for babies using the best level of evidence) individual participant data meta-analysis (IPD-MA) were to assess the effect of antenatal magnesium sulphate when given to women at risk of preterm birth on important clinical outcomes and whether treatment effects varied depending on participant and treatment factors.

**What did the researchers do and find?:**

- Five randomised trials with 5,493 women and 6,131 babies were identified as including women at risk of preterm birth who were allocated magnesium sulphate or control treatment and in which neurologic outcomes for the baby were reported and included.
- Antenatal magnesium sulphate given to women at imminent risk of preterm birth for fetal neuroprotection prevents cerebral palsy and reduces the combined risk of fetal/infant death or cerebral palsy.
- Benefit was seen regardless of the reason for preterm birth, across a range of preterm gestational ages, and with minimal variation in outcomes related to time prior to birth or dosage given.

**What do these findings mean?:**

- Antenatal magnesium sulphate for fetal neuroprotection can be recommended to be given close to planned or expected preterm birth using the smallest effective dose of 4 g with or without a 1 g/hour maintenance dose.
- Antenatal magnesium sulphate is an inexpensive, effective treatment that can reduce the burden of death and cerebral palsy in babies born very preterm.
- Widespread adoption of recommendations to use antenatal magnesium sulphate prior to preterm birth could lead to significant global health benefits.

## Introduction

Worldwide, each year, almost 15 million babies are born preterm (before 37 completed weeks of gestation)—11% of all births [1]. Babies born preterm are at greater risk of dying in early life compared with those born at term [2,3]. Preterm babies who survive have a higher risk for neurologic impairments, such as cerebral palsy, blindness, deafness, or cognitive dysfunction, and, as a result, are at greater risk of substantial disability [4]. The earlier the gestation at birth is, the greater these risks become. Global estimates suggest that up to 8% of preterm babies have neurological impairments, of which 5% are mild and 3% moderate or severe [5]. Cerebral palsy and cognitive dysfunction, either intellectual impairment or developmental delay, are the most frequent cause of neurological impairment. With the rate of preterm birth increasing in many countries, more babies are at risk of dying and, among those who survive, of having an adverse neurological outcome, making the global burden substantial [6]. Effective therapies that can reduce the risk of neurological impairments and disabilities for preterm survivors are urgently needed.

The linkage of antenatal magnesium sulphate to a reduction in cerebral palsy arose from seminal observational studies in the mid-1990s [7,8]. Several randomised trials followed, the latest reported in 2008 [9,10,11,12,13]. When the Cochrane review assessing the use of magnesium sulphate for women at risk of preterm birth for neuroprotection of the fetus was updated in 2009 [14], for the first time, a clinically important reduction in the risk of cerebral palsy was identified using aggregate data meta-analysis methods. There was a relative risk reduction of nearly a third in cerebral palsy (32%) in children born to women allocated magnesium sulphate prior to preterm birth compared with controls. Although the absolute risk reduction of 1.7% for cerebral palsy was small, it represented a huge advance in minimising a burden of illness that has so far proved very resistant to intervention. The exact mechanism by which magnesium exerts a neuroprotective effect is unclear. Magnesium sulphate can block cerebral glutamate receptors, and this may prevent posthypoxic brain injury in the perinatal period [14].

The Cochrane review was unable to provide some details needed by guideline developers, policy makers, clinicians, and pregnant women—notably, whether antenatal magnesium sulphate treatment is more effective in some women by reason of their risk of preterm birth, what the gestational age range is for maximal benefit, what dose and timing prior to birth is best, and whether maintenance treatment or retreatment is necessary [15,16]. The AMICABLE (Antenatal magnesium sulphate individual participant data international collaboration: Assessing the benefits for babies using the best level of evidence) Group was formed to undertake a meta-analysis of the individual participant data of the eligible trials. The advantages of individual participant data meta-analysis (IPD-MA) for exploring interactions between treatment and participant-level characteristics and enabling examination of differential treatment effects between subgroups are well described [17,18,19]. The objectives of the AMICABLE IPD-MA were to assess, using IPD methods and meta-analyses, the effects of administration of antenatal magnesium sulphate given to women at risk of preterm birth on important clinical outcomes.

## Methods

The Children, Youth and Women’s Health Service Research Ethics Committee, South Australia, Australia, approved the study (REC2315/10/13). The included trials had received country-specific ethical review, with individual participants providing written, informed consent. The IPD meta-analysis followed the published protocol (S2 Text) [20], and the results are reported using the Preferred Reporting Items for Systematic Review and Meta-analyses of Individual Participant Data (PRISMA-IPD Statement) checklist (S1 Text) [21].

### Specific objectives

The specific objectives of the AMICABLE IPD-MA were to assess whether the treatment effects of antenatal magnesium sulphate given to women at risk of preterm birth differ depending on the following important prespecified participant and treatment characteristics [20]:

- the reason the woman was considered to be at risk of preterm birth (such as preterm labour, hypertensive disease of pregnancy, antepartum haemorrhage, or presence of ruptured membranes);
- the primary reason antenatal magnesium sulphate treatment was given (such as neuroprotection of the fetus, pre-eclampsia, or tocolysis);
- the number of babies in utero (singleton or multiple);
- the gestational age at which antenatal magnesium sulphate treatment was given;
- the time prior to birth when antenatal magnesium sulphate treatment was given;
- the type, mode of administration, and dosage of antenatal magnesium sulphate planned and given;
- whether maintenance treatment with antenatal magnesium sulphate was planned and used; and
- whether repeat antenatal magnesium sulphate treatment was planned and used.

### Eligibility criteria

Trials in which women considered at raised risk of preterm birth (less than 37 weeks' gestation) were randomised to either magnesium sulphate or no treatment were considered eligible. Trials were included if the primary aim of the study was to prevent neurologic abnormalities in the unborn baby or if the primary aim was otherwise but early childhood neurological outcomes were reported for the infants. Quasirandom study designs were not eligible.

### Identifying studies: Information sources and search strategy

The search strategy developed by the Cochrane Pregnancy and Childbirth Group [22] was used. This identified trials from monthly searches of the Cochrane Central Register of Controlled Trials (CENTRAL); weekly searches of MEDLINE; monthly searches of CINAHL (EBSCO); hand-searches of 30 journals and the proceedings of major conferences; and weekly current awareness alerts for a further 44 journals, plus monthly BioMed Central email alerts.

Searches used the terms [antenatal or prenatal] and [magnesium] and [preterm or premature or neuroprotection or 'cerebral palsy']. The date of the last search was 28 February 2017. In addition, the World Health Organisation (WHO)/CTRP portal was accessed to identify any recently completed or ongoing trials. The date of the last search was 3 May 2017. There was no language restriction on the searches. Trialists within the AMICABLE Group were asked if they knew of any unpublished or other trials.

### Study selection processes

Eligibility for inclusion of the identified trials was assessed independently by 2 unblinded members of the AMICABLE Project Team (PFM and TKB). Any differences in opinion regarding eligibility were resolved by discussion. For the MAGPIE Trial [12], the primary aim of the study was neuroprotection of the mother with pre-eclampsia rather than neuroprotection of the fetus. Childhood neurological outcomes were available from centres selected for longer-term follow-up. Data were included from participants in the longer-term follow-up who were preterm at trial entry (<37 weeks’ gestation).

### Data collection processes and data items

The chair of the AMICABLE Project Team contacted the investigators of all eligible studies to invite them to join the AMICABLE Trialist Group and to include IPD from their trial in the meta-analysis.

Prespecified and clearly defined variables (both for participant-level and trial-level factors as well as for outcomes) were identified and confirmed by the AMICABLE Trialist Group. Investigators were asked whether these variables had been collected or could be derived for their study. A coding system was developed from these variables.

Deidentified data were collected on all women randomised. These included baseline data for descriptive purposes and analyses (reason at raised risk of preterm birth, gestational age at trial entry, plurality of the pregnancy, and expected date of birth) and details of the intervention planned and given (the date of randomisation, the allocated intervention, the type and dose of magnesium sulphate given, the mode of administration, whether a maintenance dose was given, and whether retreatment was given and its amount), together with the maternal and infant outcomes to allow the planned analyses.

The individual trial data were recoded as required and stored in a custom-designed secure database only accessible by authorised personnel from the AMICABLE Data Management Group. Trialists from the individual trials were asked to verify their own coded data prior to the analyses.

Data were checked with respect to range, internal consistency, missing or extreme values, errors, and consistency with the published reports. Trial details such as randomisation methods and intervention details were cross-checked against trial protocols, clinical record forms, and published reports. The AMICABLE biostatistician, project, and data managers liaised closely with the investigators from the individual trials to clarify any inconsistencies and missing data. Each trial was analysed individually, and the final IPD dataset generated for each trial was sent to the appropriate trial investigator for verification before synthesising into the full AMICABLE dataset. Data items collected are published elsewhere (S3 Text) [20].

### IPD integrity

#### Risk of bias assessment in individual studies

Using the Cochrane Collaboration risk of bias tool [23], the risk of bias for each study was assessed independently by 2 members of the AMICABLE Project Team (PFM and TKB), with differences resolved by discussion. Each study was judged to have a high, low, or unclear risk of bias for random sequence generation (checking for possible selection bias), allocation concealment (checking for possible selection bias), blinding of participants and personnel (checking for possible performance bias), blinding of outcome assessment (checking for possible detection bias), incomplete outcome data (checking for possible attrition bias due to the amount, nature, and handling of incomplete outcome data), selective reporting (checking for reporting bias), and other bias (checking for bias due to problems not already covered). The magnitude and direction of the bias and whether it was considered likely to affect the findings were assessed. Additional information was sought from the trialists if any aspect was unclear.

#### Specification of outcomes and effect measures

The 2 primary prespecified outcomes were a composite of death (defined as any fetal death after randomisation or death of a live-born infant before follow-up) or cerebral palsy for the infant (as defined by the trialists and categorised as mild, moderate, or severe) and, for the mother, any severe maternal outcome potentially related to treatment (defined as death, respiratory arrest, or cardiac arrest). Secondary outcomes were prespecified in the protocol (S4 Text) [20].

### Synthesis methods

A detailed statistical analysis plan was prepared by the AMICABLE Data Management Group and agreed upon by the AMICABLE Trialist Group prior to the data analyses. All analyses were based on the checked and updated IPD from all trials. All randomised participants with outcome data available were included in the analyses, which were performed on an intention-to-treat basis, according to the treatment allocation at randomisation. No changes were made to the prespecified analysis plan. Further description of the statistical methods is included in the protocol [20].

For each of the outcomes, a 1-stage approach to analysis was taken so that the IPD from all eligible trials were included in a single model. Fitting a single model for each outcome variable enabled the variation across trials to be accounted for within the model. A treatment-by-trial interaction term was tested to assess heterogeneity of treatment effect across trials. If there was excessive statistical heterogeneity in the treatment effect across trials (i.e., the trial-by-treatment interaction term was significant), then the rationale for combining trials was questioned and the source of heterogeneity explored.

Binary outcomes were analysed using log-binomial regression models, and the results are presented as relative risk RR) with 95% confidence intervals (CIs) and associated 2-sided *p*-values. Continuous outcomes were analysed using linear regression models, and the results are presented as differences in means with 95% CIs and 2-sided *p*-values. Correlations between outcomes due to multiple births were taken into account using generalised estimating equations (GEEs), when appropriate.

Any differences in treatment effect between prespecified subgroups were assessed by testing a treatment by subgroup interaction term within the model. If data were missing for an outcome, those participants were removed from the analysis for that outcome. Reasons for missingness were explored, when possible. For each outcome, if there were unbalanced or large amounts of missing endpoint data for at least 1 trial, then sensitivity analyses were undertaken to assess the impact of removing such trials from the analysis.

Categorical outcomes were analysed using proportional odds models. Analyses of raw growth measurements (head circumference, weight, and length) were adjusted for age corrected for prematurity and sex, and analyses of WHO calculated z-scores of growth measurements were unadjusted for age and sex.

Missing data were accounted for in the following 2 ways. The main method for accounting for missing data for binary outcomes was to assume that participants with no data for that outcome did not have the outcome. This is referred to as the ‘Impute “No”‘ method.

Secondly, for the primary outcome (death or CP) missing CP values at follow-up were imputed using multiple imputations (MIs). One hundred MI datasets were imputed using the Stata version 11.2 ‘ice’ (MI using chained equations) function. Counts and percentages within each treatment group for MI analyses represent average percentages in the imputed datasets. Trial-specific estimates and standard errors for imputed analyses are from MI GEE models adjusting for correlation between multiple births. Overall relative risk estimates for meta-analyses containing results from the MI models were calculated using a 2-stage aggregate meta-analysis approach with inverse variance weighting.

The following sensitivity analyses were conducted:

1. exclusion of trials that were not for the purpose of neuroprotection of the fetus
2. exclusion of trials with high rates of participant exclusions, where losses were considered to have the potential to affect the results.

## Results

### Study characteristics

**Fig 1:**
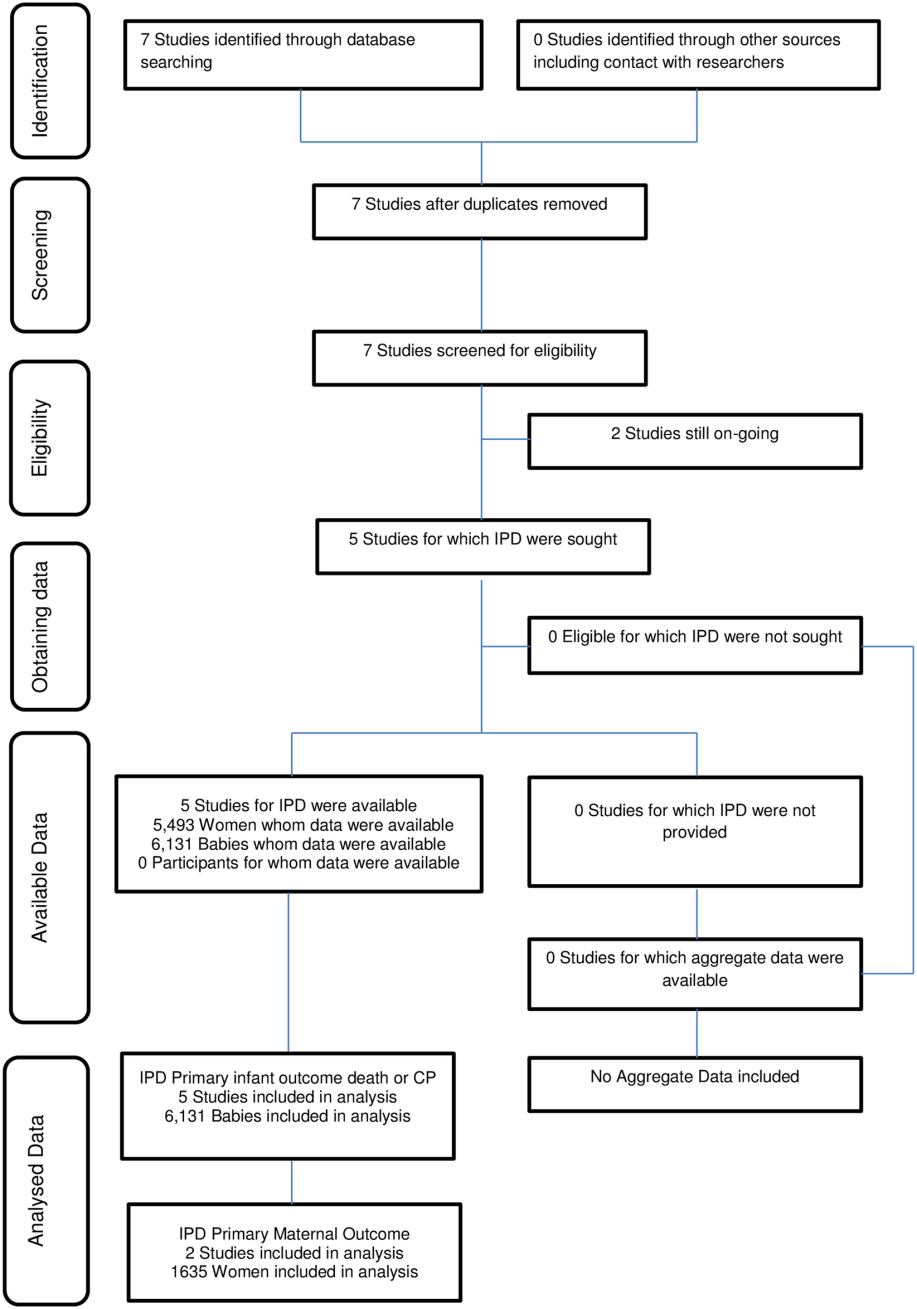
Preferred Reporting Items for Systematic Review and Meta-analyses of Individual Participant Data (PRISMA-IPD) flow diagram. Abbreviations: CP, cerebral palsy.

Seven randomised trials were identified through database searching [9,10,11,12,13,24,25], 2 of which were ongoing randomised trials [24,25]. Five randomised controlled trials were identified from the search, and each contributed data for the IPD meta-analyses (Fig 1) [9,10,11,12,13]. Details of the studies are described in Table 1.

**Table 1:** Included studies and their characteristics.

Trial Name	Participants included in IPD	Interventions	Outcomes
ACTOMgSO_4_ [9]	1,062 women (1,255 fetuses) at <30 weeks' gestation likely to give birth within 24 hours.	Active treatment: 4 g magnesium sulphate intravenously over 20 minutes and then 1 g/hour until birth or for 24 hours, whichever came first. Placebo group: equal volume of 0.9% saline.	Primary outcomes: total paediatric mortality (stillbirths, deaths up to 2 years of age, cerebral palsy, and combined outcome of death or cerebral palsy). Maternal outcomes: adverse cardiovascular and respiratory effects of infusion.
PREMAG [10]	564 women (691 fetuses) in labour at <33 weeks' gestation.	Active treatment: 4 g magnesium sulphate over 30 minutes. Placebo group: equal volumes of 0.9% saline.	Primary outcomes: infant death or white matter injury on cranial ultrasound. Secondary outcomes included follow-up of children at 2 years of age.
MAGNET [11]	149 women (166 fetuses) in preterm labour at 25–33 weeks’ gestation, with or without premature rupture of the membranes.	‘Tandem’ randomisation: (1) Eligible for aggressive tocolysis (cervix ≤ 4 cm dilation). Magnesium sulphate tocolysis: 4 g bolus and then 2–3 g/hour maintenance (*n* = 46). ‘Other’ tocolysis (*n* = 46). (2) Not eligible for tocolysis (cervix > 4 cm dilatation). Neuroprotective magnesium sulphate: 4 g bolus alone (*n* = 29). Saline control (*n* = 28).	Fetal and later mortality, IVH, cerebral palsy, and death or cerebral palsy at 18 months of age.
MAGPIE [12]	1,544 women (1,575 fetuses); a subset of women in the Magpie Trial who were <37 weeks' gestation with severe pre-eclampsia and randomised prior to birth in centres included in long-term follow-up.	Active treatment: 4 g magnesium sulphate intravenously over 10–15 minutes, followed by either 1 g/hour intravenously for 24 hours or by 5 g every 4 hours intramuscularly for 24 hours. Placebo: equal volumes of 0.9% saline.	Primary outcomes: eclampsia and death of the baby before hospital discharge. Secondary endpoints included long-term outcomes for the children. Data for paediatric mortality and cerebral palsy were provided at 18 months of age.
BEAM [13]	2,241 women (2,444 fetuses) at 24 to <32 weeks' gestation, at high risk of spontaneous birth due to ruptured membranes at 22–31 weeks' GA, or advanced preterm labour with dilatation 4–8 cm and intact membranes. Individuals were also included if an indicated preterm birth was anticipated within 24 hours.	Active treatment: 6 g magnesium sulphate intravenously over 20–30 minutes, followed by maintenance infusion of 2 g/hour. If delivery had not occurred after 12 hours and was no longer considered imminent, the infusion was discontinued and resumed when delivery threatened. Placebo group received an ‘identical-appearing placebo’.	Primary outcomes: composite of (1) stillbirth or infant death by 1 year of age or (2) moderate or severe cerebral palsy at or beyond 2 years of age (corrected).

Abbreviations: ACTOMgSO_4_, Australasian Collaborative Trial of Magnesium Sulphate; BEAM, Beneficial Effects of Antenatal Magnesium Sulfate; GA, gestational age; IPD, individual participant data; IVH, intraventricular haemorrhage; MAGNET, Magnesium and Neurologic Endpoints Trial; MAGPIE, MAGnesium sulphate for Prevention of Eclampsia; PREMAG, PREterm brain protection by MAGnesium sulphate

### IPD integrity

Overall, the risk of bias was low in the included studies, with some variation between trials (Table 2).

**Table 2:** Risk of bias within studies.

Trial	Randomisation^1^	Concealment^2^	Blinding^3^	Attrition^4^	Reporting^5^
ACTOMgSO_4_ [9]	low	low	low	low	low
PREMAG [10]	low	low	low	unclear	low
MAGNET [11]	unclear	unclear	unclear	unclear	unclear
MAGPIE [12]	low	low	low	unclear	low
BEAM [13]	low	low	low	low	low

Abbreviations: ACTOMgSO_4_, Australasian Collaborative Trial of Magnesium Sulphate; BEAM, Beneficial Effects of Antenatal Magnesium Sulfate; MAGNET, Magnesium and Neurologic Endpoints Trial; MAGPIE, MAGnesium sulphate for Prevention of Eclampsia; PREMAG, PREterm brain protection by MAGnesium sulphate.

^1^Random sequence generation.

^2^Allocation concealment.

^3^Blinding of performance and detection.

^4^Incomplete outcome data.

^5^Selective reporting.

### Risk of bias across studies

There was a generally low risk of bias across the studies, except for 3 of the 5 studies in which the risk of bias associated with attrition was rated as unclear.

### Primary outcomes

#### A. Death or cerebral palsy

There was no statistically significant effect of antenatal treatment with magnesium sulphate on the composite outcome death or cerebral palsy in infants in the combined analyses (Table 3).

**Table 3:** Death or cerebral palsy* (all trials).

Trial	MgSO_4_	Control	Relative risk	95% confidence interval
ACTOMgSO_4_ [9]	123/629 (19.6%)	150/626 (24.0%)	0.83	0.67–1.04
PREMAG [10]	56/353 (15.9%)	68/338 (20.1%)	0.76	0.54–1.07
MAGNET [11]	13/86 (15.1%)	4/80 (5.0%)	2.80	0.94–8.34
MAGPIE [12]	202/790 (25.6%)	182/ 785 (23.2%)	1.07	0.90–1.28
BEAM [13]	148/1,188 (12.5%)	173/1,256 (13.8%)	0.91	0.85–1.05
Overall^†^	542/3,046 (17.8%)	577/3,085 (18.7%)	0.94	0.85–1.05

Abbreviations: ACTOMgSO_4_, Australasian Collaborative Trial of Magnesium Sulphate; BEAM, Beneficial Effects of Antenatal Magnesium Sulfate; MAGNET, Magnesium and Neurologic Endpoints Trial; MAGPIE, MAGnesium sulphate for Prevention of Eclampsia; PREMAG, PREterm brain protection by MAGnesium.

*Available data (participants were included if either death or cerebral palsy [CP] outcome known).

^†^*p*-value for heterogeneity = 0.07 (the heterogeneity *p*-value for 1-stage analyses is from Wald chi-square tests for the interaction between treatment and trial in a generalising estimating equation [GEE] model).

In the prespecified sensitivity analysis of the 4 trials with fetal neuroprotective intent, there was a statistically significant reduction in the risk of death or cerebral palsy (Table 4) with no significant heterogeneity (*p* = 0.28). The number needed to treat (NNT) to benefit was 41 women/babies to prevent 1 baby from either dying or having cerebral palsy (CP).

**Table 4:** Death or cerebral palsy (sensitivity analysis: trials with a primary intent of fetal neuroprotection)*.

Trial	MgSO_4_	Control	Relative risk	95% confidence interval
ACTOMgSO_4_ [9]	123/629 (19.6%)	150/626 (24.0%)	0.83	0.67–1.04
PREMAG [10]	56/353 (15.9%)	68/338 (20.1%)	0.76	0.54–1.07
MAGNET [11]	5/28 (17.9%)	1/30 (3.3%)	5.36	0.66–43.20
BEAM [13]	148/1,188 (12.5%)	173/1,256 (13.8%)	0.91	0.74–1.12
Overall	332/2,198 (15.1%)	392/2,550 (17.4%)	0.86	0.75–0.99

Abbreviations: ACTOMgSO_4_, Australasian Collaborative Trial of Magnesium Sulphate; BEAM, Beneficial Effects of Antenatal Magnesium Sulfate; MAGNET, Magnesium and Neurologic Endpoints Trial; MgSO_4_, magnesium sulphate; PREMAG, PREterm brain protection by MAGnesium sulphate.

*Available data (participants were included if either death or cerebral palsy [CP] outcome known).

#### B. Severe adverse maternal events related to treatment

There were no events for the primary outcome of severe adverse maternal events related to treatment (including death or respiratory or cardiac arrest) and, accordingly, no events for maternal death or for severe maternal adverse events reported separately (total of 1,635 women: [9,10]).

#### C. Paediatric mortality

For the primary paediatric outcome of mortality, the overall mortality rates were 14.3% for babies exposed to magnesium sulphate and 13.6% for those exposed to the control treatment, not a statistically significant effect (Table 5).

**Table 5:** Paediatric death (fetal, neonatal, or later death) at any time* (all trials).

Trial	MgSO_4_	Control	Relative risk	95% confidence interval
ACTOMgSO_4_ [9]	87/629 (13.8%)	108/626 (17.3%)	0.81	0.62–1.07
PREMAG [10]	34/353 (9.6%)	38/338 (11.2%)	0.83	0.52–1.32
MAGNET [11]	10/86 (11.6%)	1/80 (1.3%)	8.11	1.05–62.69
MAGPIE [12]	200/790 (25.3%)	177/785 (22.5%)	1.09	0.91–1.30
BEAM^‡^ [13]	105/1,188 (8.8%)	97/1,256 (7.7%)	1.15	0.88–1.51
Overall^†^	436/3,046 (14.3%)	421/3,085 (13.6%)	1.03	0.91–1.17

Abbreviations: ACTOMgSO_4_, Australasian Collaborative Trial of Magnesium Sulphate; BEAM, Beneficial Effects of Antenatal Magnesium Sulfate; MAGNET, Magnesium and Neurologic Endpoints Trial; MAGPIE, MAGnesium sulphate for Prevention of Eclampsia; MgSO_4_, magnesium sulphate; PREMAG, PREterm brain protection by MAGnesium sulphate.

*Available data.

^‡^The BEAM trial only recorded deaths in the first 12 months of life

^†^*p*-value for heterogeneity = 0.07 (the heterogeneity *p*-value for 1-stage analyses is from Wald chi-square tests for the interaction between treatment and trial in a generalising estimating equation [GEE] model).

#### D. Cerebral palsy

There was a strong protective effect of magnesium sulphate on the rate of CP, both for all studies (NNT to benefit 46 babies) (Table 6) and for the analysis limited to the fetal neuroprotective studies only (NNT to benefit 42 babies) (Table 7).

**Table 6:** Cerebral palsy (as defined by trialists)* (all trials).

Trial	MgSO_4_	Control	Relative risk	95% confidence interval
ACTOMgSO_4_ [9]	36/533 (6.8%)	42/513 (8.2%)	0.83	0.54–1.28
PREMAG [10]	22/313 (7.0%)	30/293 (10.2%)	0.68	0.40–1.17
MAGNET [11]	3/60 (5.0%)	3/62 (4.8%)	1.03	0.22–4.94
MAGPIE [12]	2/236 (0.8%)	5/255 (2.0%)	0.43	0.08–2.21
BEAM [13]	43/1,133 (3.8%)	77/1,203 (6.4%)	0.59	0.41–0.86
Overall^†^	106/2,275 (4.7%)	157/2,326 (6.7%)	0.68	0.54–0.87

Abbreviations: ACTOMgSO_4_, Australasian Collaborative Trial of Magnesium Sulphate; BEAM, Beneficial Effects of Antenatal Magnesium Sulfate; MAGNET, Magnesium and Neurologic Endpoints Trial; MAGPIE, MAGnesium sulphate for Prevention of Eclampsia MgSO_4_, magnesium sulphate; PREMAG, PREterm brain protection by MAGnesium sulphate.

*Available data (participants were included if the cerebral palsy [CP] outcome was known).

^†^*p*-value for heterogeneity = 0.74 (the heterogeneity *p*-value for 1-stage analyses is from Wald chi-square tests for the interaction between treatment and trial in a generalising estimating equation [GEE] model).

**Table 7:** Cerebral palsy (sensitivity analysis: trials with a primary intent of fetal neuroprotection)*.

Trial	MgSO_4_	Control	Relative risk	95% confidence interval
ACTOMgSO_4_ [9]	36/533 (6.8%)	42/513 (8.2%)	0.83	0.54–1.28
PREMAG [10]	22/313 (7.0%)	30/293 (10.2%)	0.68	0.40–1.17
MAGNET [11]^‡^	3/23 (13.0%)	0/23 (0%)	–	–
BEAM [13]	43/1,133 (3.8%)	77/1,203 (6.4%)	0.59	0.41–0.86
Overall^†^	101/1,979 (5.1%)	149/2,009 (7.4%)	0.68	0.53–0.87

Abbreviations: ACTOMgSO_4_, Australasian Collaborative Trial of Magnesium Sulphate; BEAM, Beneficial Effects of Antenatal Magnesium Sulfate; MAGNET, Magnesium and Neurologic Endpoints Trial; MgSO_4_, magnesium sulphate; PREMAG, PREterm brain protection by MAGnesium sulphate. The denominators exclude the deaths of infants/children.

*Available data (participants were included if the cerebral palsy [CP] outcome was known).

^‡^The MAGNET neuroprotective study is shown here but did not contribute to the analyses (no events in the control group).

^†^*p*-value for heterogeneity = 0.49 (the heterogeneity *p*-value for 1-stage analyses is from Wald chi-square tests for the interaction between treatment and trial in a generalising estimating equation [GEE] model).

Overall, there were significant reductions in the rates of both moderate and severe CP combined (event rates 2.12% MgSO_4_, 3.36% controls; RR 0.63, 95% CI 0.44 to 0.90) and severe CP alone (event rates 0.81% MgSO_4_, 1.50% controls; RR 0.54, 95% CI 0.30 to 0.94), with no significant heterogeneity among trials for either outcome.

### Maternal secondary outcomes

Of the maternal secondary outcomes, the only statistically significant effect was an increase in adverse events leading to stopping treatment in the magnesium sulphate group (Table 8). There were no clear differences in infectious morbidity (19.1% magnesium sulphate versus 18.8% control), mode of birth by caesarean (48% versus 46.3%), or postpartum haemorrhage (28.1% versus 28.1%).

**Table 8:** Maternal secondary outcomes*.

Outcome	Studies contributing data	MgSO_4_	Control	RR	LCL	UCL	*p*^†^
Dichotomous							
Adverse event leading to stopping treatment	9,10,11,12,13	115/2,310 (5.0%)	58/2,337 (2.5%)	1.95	1.44	2.65	0.11
Intrapartum fever treated with antibiotics	9,11	237/603 (39.3%)	227/592 (38.3%)	1.05	0.94	1.18	0.37
Clinical chorioamnionitis during labour	9,11,13	323/1,695 (19.1%)	326/1,738 (18.8%)	1.00	0.87	1.14	0.92
Caesarean delivery	9,10,11,12,13	1,310/2,727 (48.0%)	1,282/2,766 (46.3%)	1.04	0.98	1.10	0.38
Postpartum haemorrhage	9,10,11,12	410/1,457 (28.1%)	410/1,458 (28.1%)	1.01	0.90	1.13	0.99

Abbreviations: LCL, lower confidence limit; UCL, upper confidence limit. The trials included are as follows: 9 = Australasian Collaborative Trial of Magnesium Sulphate (ACTOMgSO_4_), 10 = PREterm brain protection by MAGnesium sulphate (PREMAG), 11 = Magnesium and Neurologic Endpoints Trial (MAGNET), 12 = MAGnesium sulphate for Prevention of Eclampsia (MAGPIE), and 13 = Beneficial Effects of Antenatal Magnesium Sulfate (BEAM).

*Overall results from 1-stage individual participant data (IPD).

^†^*p*-values for heterogeneity (the heterogeneity *p*-value for 1-stage analyses is from Wald chi-square tests for the interaction between treatment and trial in a generalising estimating equation [GEE] model).

### Neonatal secondary outcomes

No statistically significant differences or any significant heterogeneity were seen in any of the analyses for the other neonatal morbidity dichotomous outcomes reported or defined by the trialists, including Apgar score at 5 minutes < 7, active resuscitation at birth, use of ongoing respiratory support after birth, any intraventricular haemorrhage (IVH), severe IVH (grade 3 or 4), cystic periventricular leucomalacia (PVL), neonatal convulsions, neonatal encephalopathy, chronic lung disease/bronchopulmonary dysplasia (BPD), posthaemorrhagic hydrocephalus or ventriculomegaly, proven systemic infection, necrotising enterocolitis (NEC), patent ductus arteriosus (PDA) requiring treatment, any retinopathy of prematurity (ROP), or severe neonatal adverse outcome (death, BPD, PDA, NEC, stage 3 or worse ROP, or grade 3 or 4 IVH); or the continuous outcomes (weight, head circumference, or length at birth) (Table 9). There was, however, a slightly lower birthweight z-score (mean difference −0.05, 95% CI −0.10 to −0.00; *p* = 0.04), a result obtained with no significant heterogeneity among the trials (*p* = 0.82) (Table 9).

**Table 9:** Neonatal secondary outcomes*.

**Outcome**	**Studies contributing data**	**MgSO_4_**	**Control**	**RR**	**LCL**	**UCL**	***p***^†^
**Dichotomous**							
Apgar score < 7 at 5 minutes	9,10,11,12,13	414/2,903 (14.3%)	420/2,961 (14.2%)	1.01	0.89	1.14	0.38
Active resuscitation at birth	9,10,12,13	1,218/2,836 (42.9%)	1,284/2,891 (44.4%)	0.99	0.96	1.02	0.46
Respiratory distress syndrome	9,10,11,13	1,288/2,215 (58.1%)	1,325/2,268 (58.4%)	1.01	0.97	1.05	0.46
Ongoing use of respiratory support	9,10,11,12,13	1,544/2,667 (57.9%)	1,612/2,710 (59.5%)	1.00	0.97	1.03	0.10
Chronic lung disease/BPD	9,10,11,13	442/2,223 (19.9%)	420/2,273 (18.5%)	1.08	0.96	1.22	0.56
Neonatal convulsions	9,10,11,12,13	68/2,884 (2.4%)	89/2,930 (3.0%)	0.75	0.54	1.03	0.91
Any IVH	9,10,11,13	461/2,149 (21.5%)	482/2,202 (21.9%)	0.98	0.87	1.09	0.37
Grade 3 or 4 IVH	9,10,11,13	96/2,149 (4.5%)	114/2,202 (5.2%)	0.83	0.63	1.09	0.48
Cystic PVL	9,10,11,13	70/2,044 (3.4%)	76/2,089 (3.6%)	0.91	0.66	1.25	0.90
Posthaemorrhagic hydrocephaly or ventriculomegaly	9,10,11	57/1,000 (5.7%)	53/976 (5.4%)	1.06	0.73	1.53	0.78
Proven neonatal systemic infection	9,10,11,13	432/2,213 (19.5%)	417/2,266 (18.4%)	1.06	0.94	1.20	0.47
Necrotising enterocolitis	9,10,11,13	155/2,142 (7.2%)	131/2,190 (6.0%)	1.22	0.97	1.53	0.56
Patent ductus arteriosus requiring treatment	9,10,11,13	425/2,214 (19.2%)	432/2,264 (19.1%)	1.03	0.91	1.16	0.50
Any retinopathy of prematurity	9,10,11,13	470/1,977 (23.8%)	469/2,023 (23.2%)	1.03	0.92	1.15	0.75
Severe neonatal adverse outcome^#^	9,10,11,13	1,021/2,225 (45.9%)	1,011/2,282 (44.3%)	1.03	0.97	1.10	0.44
**Continuous**		**Mean (SE)**	**Mean (SE)**	**Mean difference**	**LCL**	**UCL**	***p***^†^
Gestational age at birth (weeks)	9,10,11,12,13	30.0 (29.8, 30.1)^‡^ (*n* = 2,996)	29.9 (29.8, 30.1)^‡^ (*n* = 3,057)	0.0	−0.1	0.2	0.95
Birth weight z-score	9,10,11,12,13	−0.25 (0.02) (*n* = 2,987)	−0.19 (0.02) (*n* = 3,047)	−0.05	−0.10	−0.00	0.82
Birth weight (g)^§^	9,10,11,12,13	1,459 (12) (*n* = 2,987)	1,471 (12) (n = 3,057)	−10	−41	21	0.96
Birth head circumference (cm)^§^	9,10,11,13	26.8 (0.1) (*n* = 2,038)	26.8 (0.1) (n = 2,091)	0.0	−0.2	0.2	0.87
Birth length (cm)^§^	9,10,11,13	38.4 (0.1) (*n* = 1,954)	38.5 (0.1) (*n* = 2,009)	−0.0	−0.3	0.3	0.85

Abbreviations: BPD, bronchopulmonary dysplasia; IVH, intraventricular haemorrhage; LCL, lower confidence limit; PVL, periventricular leucomalacia; RR, relative risk; SE, standard error; UCL, upper confidence limit. The trials included are as follows: 9 = Australasian Collaborative Trial of Magnesium Sulphate (ACTOMgSO_4_), 10 = PREterm brain protection by MAGnesium sulphate (PREMAG), 11 = Magnesium and Neurologic Endpoints Trial (MAGNET), 12 = MAGnesium sulphate for Prevention of Eclampsia (MAGPIE), and 13 = Beneficial Effects of Antenatal Magnesium Sulfate (BEAM).

*Overall results from 1-stage individual participant data (IPD).

^#^Any of the following: death, chronic lung disease, patent ductus arteriosus requiring treatment, neonatal encephalopathy, necrotising enterocolitis, stage 3 or worse retinopathy of prematurity, or grade 3 or 4 intraventricular haemorrhage (IVH).

^†^Heterogeneity *p*-values for 1-stage analyses.

^§^Adjusted for age and sex.

### Paediatric secondary outcomes

The meta-analysis found no evidence of statistically significant differences or significant heterogeneity for any of the analyses for the follow-up outcomes reported or defined by the trialists. Of note, weight z-scores at follow-up did not clearly differ between groups (Table 10).

**Table 10:** Paediatric secondary outcomes*.

**Outcome**	**Studies contributing data**	**MgSO_4_**	**Control**	**RR**	**LCL**	**UCL**	***p***^†^
**Dichotomous follow-up outcomes**							
Blind	9,10,12,13	92/1,332 (6.9%)	128/1,383 (9.3%)	0.83	0.65	1.06	0.99
Deaf	9,10,11,12,13	83/1,094 (7.6%)	83/1,167 (7.1%)	1.10	0.83	1.47	0.99
Developmental delay: any	9,10,12,13	674/1,985 (34.0%)	706/2,027 (34.8%)	0.99	0.91	1.08	0.86
Gross motor dysfunction: any	9,10,13	239/1,707 (14.0%)	279/1,715 (16.3%)	0.85	0.72	1.00	0.47
Gross motor dysfunction: moderate or severe	9,10,13	55/1,707 (3.2%)	70/1,715 (4.1%)	0.78	0.55	1.11	0.69
Neurosensory disability: any	9,10,11,12,13	795/2,283 (34.8%)	851/2,339 (36.4%)	0.98	0.90	1.05	0.65
Major neurosensory disability	9,10,12,13	404/2,222 (18.2%)	427/2,277 (18.8%)	1.00	0.88	1.13	0.43
Death or any neurosensory disability	9,10,11,12,13	1,228/3,046 (40.3%)	1,271/3,085 (41.2%)	0.99	0.93	1.05	0.09
Death or major neurosensory disability	9,10,11,12,13	839/3,046 (27.5%)	848/3,085 (27.5%)	1.01	0.93	1.10	0.13
Death or moderate/severe motor dysfunction	9,10,13	281/2,170 (12.9%)	312/2,220 (14.1%)	0.91	0.78	1.06	0.40
**Continuous follow-up outcomes**		**Mean (SE)**	**Mean (SE)**	**Mean difference**	**LCL**	**UCL**	***p***^†^
Weight z-score^§^	9,10	−0.33 (0.04) (*n* = 823)	−0.38 (0.04) (*n* = 781)	0.04	−0.09	0.16	0.55
Length/height z-score	9,10	−0.72 (0.05) (*n* = 708)	−0.66 (0.06) (*n* = 653)	−0.06	−0.21	0.09	0.34
Head circumference^§^	9,10	48.6 (0.07) (*n* = 779)	48.5 (0.07) (*n* = 732)	0.1	−0.1	0.3	0.24

Abbreviations: LCL, lower confidence limit; RR, relative risk; SE, standard error; UCL, upper confidence limit. The trials included are as follows: 9 = Australasian Collaborative Trial of Magnesium Sulphate (ACTOMgSO_4_), 10 = PREterm brain protection by MAGnesium sulphate (PREMAG), 11 = Magnesium and Neurologic Endpoints Trial (MAGNET), 12 = MAGnesium sulphate for Prevention of Eclampsia (MAGPIE), and 13 = Beneficial Effects of Antenatal Magnesium Sulfate (BEAM).

*Overall results from 1-stage individual participant data (IPD).

^†^Heterogeneity *p*-values for 1-stage analyses.

^§^Adjusted for age and sex.

### Subgroup analyses

As there were no events, the planned subgroup analyses for severe maternal adverse outcomes potentially related to treatment were not possible. Not all trials had data available to contribute to all subgroup analyses.

#### A. Primary reason pregnancy was considered to be at raised risk of preterm birth

Among the 4 trials able to contribute data to this analysis, there were no clear differences in treatment effects among the subgroups considered by individual causes for the high-risk pregnancy (Table 11).

**Table 11:** Treatment effects by the individual reasons why the pregnancy was considered to be high risk.

Outcome	Subgroup	Included trials	MgSO_4_	Control	RR	LCL	UCL	*p*^†^
Death or CP	Preeclampsia	9,12	215/883 (24.3%)	195/866 (22.5%)	1.06	0.89	1.26	0.15
	Preterm labour	9,10,11	91/477 (19.1%)	109/484 (22.5%)	0.82	0.63	1.06	–
	Chorioamnionitis	9,10	32/133 (24.1%)	32/141 (22.7%)	1.06	0.68	1.66	–
	APH	10	8/47 (17.0%)	13/40 (32.5%)	0.53	0.23	1.24	–
	PROM < 24 hours	9,10	3/31 (9.7%)	6/34 (17.6%)	0.64	0.17	2.42	–
	PROM ≥ 24 hours	10,11	25/144 (17.4%)	12/122 (9.8%)	1.69	0.87	3.25	–
	Other	9,10	11/63 (17.5%)	18/55 (32.7%)	0.53	0.28	1.02	–
Death (at any time)	Preeclampsia	9,12	207/883 (23.4%)	187/866 (21.6%)	1.07	0.89	1.27	0.34
	Preterm labour	9,10	58/438 (13.2%)	72/443 (16.3%)	0.77	0.55	1.08	–
	Chorioamnionitis	9,10	26/133 (19.5%)	25/141 (17.7%)	1.17	0.70	1.95	–
	APH	10	6/47 (12.8%)	7/40 (17.5%)	0.85	0.28	2.62	–
	PROM < 24 hours	9,10	1/31 (3.2%)	4/34 (11.8%)	0.30	0.03	2.68	–
	PROM ≥ 24 hours	10,11	11/144 (7.6%)	5/122 (4.1%)	1.71	0.59	4.90	–
	Other	9,10	8/63 (12.7%)	14/55 (25.5%)	0.53	0.24	1.17	–
Cerebral palsy*	Preeclampsia	9,12	8/320 (2.50%)	8/325 (2.5%)	0.95	0.37	2.44	0.48
	Preterm labour	9,10,11	27/401 (6.7%)	37/395 (9.4%)	0.71	0.45	1.14	–
	Chorioamnionitis	9,10	6/107 (5.6%)	7/116 (6.0%)	0.87	0.31	2.49	–
	APH	10	2/41 (4.9%)	6/32 (18.8%)	0.26	0.06	1.19	–
	PROM ≥ 24 hours	10	11/96 (11.5%)	7/80 (8.8%)	1.27	0.51	3.20	–
Death or major neurosensory disability	Preeclampsia	9,12	227/883 (25.7%)	207/866 (23.9%)	1.06	0.90	1.25	0.53
	Preterm labour	9,10	112/438 (25.6%)	126/443 (28.4%)	0.88	0.70	1.11	–
	Chorioamnionitis	9,10	35/133 (26.3%)	32/141 (22.7%)	1.24	0.80	1.91	–
	APH	9,10	9/47 (19.1%)	9/43 (20.9%)	1.14	0.44	2.92	–
	PROM < 24 hours	9,10	4/31 (12.9%)	11/34 (32.4%)	0.50	0.18	1.38	–
	PROM ≥ 24 hours	9	12/105 (11.4%)	9/86 (10.5%)	1.05	0.45	2.41	–
	Other	9,10	16/63 (25.4%)	21/55 (38.2%)	0.70	0.41	1.19	–

Abbreviations: APH, antepartum haemorrhage; CP, cerebral palsy; LCL, lower confidence limit; PROM, prelabour rupture of membranes; RR, relative risk; UCL, 95% upper confidence limit. The trials included are as follows: 9 = Australasian Collaborative Trial of Magnesium Sulphate (ACTOMgSO_4_), 10 = PREterm brain protection by MAGnesium sulphate (PREMAG), 11 = Magnesium and Neurologic Endpoints Trial (MAGNET), 12 = MAGnesium sulphate for Prevention of Eclampsia (MAGPIE) and 13 = Beneficial Effects of Antenatal Magnesium Sulfate (BEAM).

*Some subgroups were not included as no events were observed.

^†^*p*-values for heterogeneity (the heterogeneity *p*-value for 1-stage analyses is from Wald chi-square tests for the interaction between treatment and subgroup in a generalising estimating equation [GEE] model).

#### B. Purpose of treatment: Neuroprotection of the fetus versus other purpose

There was a significant difference in the magnitude of the treatment effect for death or CP for studies with the intent of fetal neuroprotection compared with studies with a different purpose. A significant reduction in death or CP was seen in the ‘neuroprotection’ group, but not in the ‘other’ reason group. There were no clear differences in treatment effects for the other outcomes (Table 12).

**Table 12:** Treatment effects among the subgroups considered by the purpose of the treatment.

Outcome	Subgroup	Included trials	MgSO_4_	Control	RR	LCL	UCL	*p*^†^
Death or CP	Neuroprotection fetus	9,10,11,13	332/2,198 (15.1%)	392/2,250 (17.4%)	0.86	0.75	0.99	0.04
	Other	11,12	210/848 (24.8%)	185/835 (22.2%)	1.08	0.91	1.29	
Death (at any time)	Neuroprotection fetus	9,10,11,13	228/2,198 (10.4%)	244/2,250 (10.8%)	0.95	0.80	1.13	0.19
	Other	11,12	208/848 (24.5%)	177/835 (21.2%)	1.12	0.93	1.33	
Cerebral palsy	Neuroprotection fetus	9,10,11,13	104/2,002 (5.2%)	149/2,032 (7.3%)	0.70	0.55	0.90	0.22
	Other	11,12	2/273 (0.7%)	8/294 (2.7%)	0.27	0.06	1.24	
Death or major neurosensory disability	Neuroprotection fetus	9,10,11,13	622/2,198 (28.3%)	659/2,250 (29.3%)	0.99	0.90	1.09	0.29
	Other	11,12	217/848 (25.6%)	189/835 (22.6%)	1.10	0.92	1.30	

Abbreviations: CP, cerebral palsy; LCL, lower confidence limit; RR, relative risk; UCL, upper confidence limit. The trials included are as follows: 9 = Australasian Collaborative Trial of Magnesium Sulphate (ACTOMgSO_4_), 10 = PREterm brain protection by MAGnesium sulphate (PREMAG), 11 = Magnesium and Neurologic Endpoints Trial (MAGNET), 12 = MAGnesium sulphate for Prevention of Eclampsia (MAGPIE), and 13 = Beneficial Effects of Antenatal Magnesium Sulfate (BEAM).

^†^*p*-values for heterogeneity (the heterogeneity *p*-value for 1-stage analyses is from Wald chi-square tests for the interaction between treatment and subgroup in a generalising estimating equation [GEE] model).

#### C. Multiple birth

There were no clear differences in treatment effects among the subgroups considered by multiple birth (Table 13).

**Table 13:** Treatment effects among the subgroups considered by multiple birth.

Outcome	Subgroup	Included trials	MgSO_4_ *n*/*N* (%)	Control *n*/*N* (%)	RR	LCL	UCL	*p*^†^
Death or CP	Singleton	9,10,11,12,13	429/2,446 (17.5%)	462/2,472 (18.7%)	0.94	0.83	1.05	0.55
	Multiple	9,10,11,13	90/517 (17.4%)	109/541 (20.1%)	0.86	0.65	1.16	–
Death (at any time)	Singleton	9,10,11,12,13	347/2,446 (14.2%)	345/2,472 (14.0%)	1.01	0.88	1.16	0.88
	Multiple	9,10,11,13	66/517 (12.8%)	70/541 (12.9%)	0.99	0.68	1.43	–
Cerebral palsy	Singleton	9,10,11,12,13	82/1,783 (4.6%)	117/1,804 (6.5%)	0.70	0.53	0.92	0.68
	Multiple	9,10,13	24/453 (5.3%)	40/474 (8.4%)	0.64	0.38	1.07	–
Death or major neurosensory disability	Singleton	9,10,11,12,13	674/2,446 (27.6%)	672/2,472 (27.2%)	1.02	0.93	1.11	0.30
	Multiple	9,10,13	136/496 (27.4%)	169/526 (32.1%)	0.91	0.73	1.14	–

Abbreviations: CP, cerebral palsy; LCL, lower confidence limit; RR, relative risk; UCL, upper confidence limit. The trials included are as follows: 9 = Australasian Collaborative Trial of Magnesium Sulphate (ACTOMgSO_4_), 10 = PREterm brain protection by MAGnesium sulphate (PREMAG), 11 = Magnesium and Neurologic Endpoints Trial (MAGNET), 12 = MAGnesium sulphate for Prevention of Eclampsia (MAGPIE), and 13 = Beneficial Effects of Antenatal Magnesium Sulfate (BEAM).

^†^*p*-values for heterogeneity (the heterogeneity *p*-value for 1-stage analyses is from Wald chi-square tests for the interaction between treatment and subgroup in a generalising estimating equation [GEE] model).

#### D. Gestational age at randomisation

Inclusion criteria for gestational age at trial entry varied across trials. Several different, prespecified combinations of gestational age groupings were considered. No obvious monotonic trends were seen for any of the major outcomes when categorised by gestational age when first treated, and no statistically significant heterogeneity was observed among any subgroups (Table 14, S2 Table).

**Table 14:** Treatment effects among the subgroups of gestational age when treatment first given.

Outcome	GA (weeks)	Included trials	MgSO_4_	Control	RR	LCL	UCL	*p*^†^
Death or CP	<26	9,10,11,12,13	206/527 (39.1%)	223/531 (42.0%)	0.99	0.87	1.12	0.78^‡^
	26–27	9,10,11,12,13	107/512 (20.9%)	132/569 (23.2%)	0.91	0.75	1.12	–
	28–29	9,10,11,12,13	98/732 (13.4%)	98/706 (13.9%)	1.00	0.80	1.26	–
	30–31	10,11,12,13	60/629 (9.54%)	58/643 (9.02%)	1.04	0.75	1.44	–
	32+	10,11,12	71/641 (11.1%)	66/632 (10.4%)	1.06	0.78	1.46	–
Death (at any time)	<26	9,10,11,12,13	169/527 (32.1%)	169/531 (31.8%)	1.06	0.92	1.22	0.86^‡^
	26–27	9,10,11,12,13	77/512 (15.0%)	89/569 (15.6%)	1.00	0.78	1.26	–
	28–29	9,10,11,12,13	71/732 (9.7%)	60/706 (8.5%)	1.14	0.87	1.49	–
	30–31	10,11,12,13	50/629 (8.0%)	42/643 (6.5%)	1.18	0.82	1.70	–
	32+	10,11,12	69/641 (10.8%)	61/632 (9.7%)	1.12	0.81	1.54	–
Cerebral palsy	<28	9,10,11,13	67/837 (8.0%)	98/876 (11.2%)	0.69	0.52	0.93	0.85
	28–31	9,10,11,12,13	37/1150 (3.2%)	54/1154 (4.7%)	0.69	0.45	1.05	–
	32+	10,12	2/253 (0.8%)	5/264 (1.9%)	0.42	0.08	2.14	–
Death or major neurosensory disability	<26	9,10,11,12,13	305/527 (57.9%)	298/531 (56.1%)	1.05	0.95	1.16	0.72
	26–27	9,10,11,12,13	154/512 (30.1%)	194/569 (34.1%)	0.93	0.79	1.10	–
	28–29	9,10,11,12,13	179/732 (24.5%)	161/706 (22.8%)	1.07	0.90	1.28	–
	30–31	10,11,12,13	125/629 (19.9%)	127/643 (19.8%)	1.04	0.83	1.30	–
	32+	10,11,12,13	76/645 (11.8%)	68/634 (10.7%)	1.10	0.81	1.49	–

Abbreviations: CP, cerebral palsy; GA, gestational age; LCL, lower confidence limit; RR, relative risk; UCL, upper confidence limit. The trials included are as follows: 9 = Australasian Collaborative Trial of Magnesium Sulphate (ACTOMgSO_4_), 10 = PREterm brain protection by MAGnesium sulphate (PREMAG), 11 = Magnesium and Neurologic Endpoints Trial (MAGNET), 12 = MAGnesium sulphate for Prevention of Eclampsia (MAGPIE), and 13 = Beneficial Effects of Antenatal Magnesium Sulfate (BEAM).

^†^*p*-values for heterogeneity (the heterogeneity *p*-value for 1-stage analyses is from Wald chi-square tests for the interaction between treatment and subgroup in a generalising estimating equation [GEE] model).

^‡^Heterogeneity *p*-value derived from logistic model due to nonconvergence of model containing treatment x subgroup interaction term.

#### E. Time from first treatment until birth

Several different, prespecified combinations of time from first treatment to birth were considered. No obvious linear trends were seen for any of the major outcomes when categorised by time from when first treated until birth, and no significant heterogeneity was observed among any subgroups (Table 15 and S3 Table).

**Table 15:** Treatment effects by subgroups according to the time from the start of treatment until birth.

Outcome	Time (hours)	Included trials	MgSO_4_	Control	RR	LCL	UCL	*p*^†^
Death or CP	0 to <4	9,10,11,12	141/808 (17.5%)	157/822 (19.1%)	0.91	0.74	1.13	0.11
	4 to <8	9,10,12	35/195 (17.9%)	27/191 (14.1%)	1.24	0.77	1.99	–
	8 to <12	9,10,12	15/95 (15.8%)	29/84 (34.5%)	0.49	0.27	0.87	–
	12 to <24	9,10,12	49/193 (25.4%)	45/182 (24.7%)	1.00	0.70	1.42	–
	24 to <36	9,12	25/70 (35.7%)	15/57 (26.3%)	1.34	0.78	2.29	–
	36+	9,11,12	117/446 (26.2%)	116/394 (29.4%)	0.92	0.73	1.14	–
Death (at any time)	0 to <4	9,10,11,12	101/808 (12.5%)	104/822 (12.7%)	1.01	0.77	1.32	0.23
	4 to <8	9,10,12	26/195 (13.3%)	20/191 (10.5%)	1.30	0.73	2.31	–
	8 to <12	9,10,12	15/95 (15.8%)	26/84 (31.0%)	0.55	0.31	1.00	–
	12 to <24	9,11,12	43/193 (22.3%)	41/182 (22.5%)	0.96	0.66	1.40	–
	24 to <36	9,12	24/70 (34.3%)	14/57 (24.6%)	1.38	0.79	2.41	–
	36+	9,11,12	112/446 (25.1%)	110/394 (27.9%)	0.93	0.74	1.17	–
Cerebral palsy	0 to <4	9,10,11	40/558 (7.2%)	53/551 (9.6%)	0.75	0.50	1.12	0.77
	4 to <12	9,10,12	9/186 (4.8%)	10/162 (6.2%)	0.71	0.30	1.68	–
	12+	9,10,11,12	13/320 (4.1%)	11/282 (3.9%)	1.13	0.47	2.68	–
Death or major neurosensory disability	0 to <4	9,10,11,12	164/808 (20.3%)	173/822 (21.0%)	1.00	0.82	1.21	0.24
	4 to <8	9,10,12	44/195 (22.6%)	30/191 (15.7%)	1.32	0.86	2.03	–
	8 to <12	9,10,12	20/95 (21.1%)	30/84 (35.7%)	0.64	0.39	1.05	–
	12 to <24	9,11,12	52/193 (26.9%)	48/182 (26.4%)	0.99	0.70	1.39	–
	24 to <36	9,12	29/70 (41.4%)	18/57 (31.6%)	1.27	0.79	2.03	–
	36+	9,11,12	126/446 (28.3%)	125/394 (31.7%)	0.94	0.76	1.16	–

Abbreviations: CP, cerebral palsy; LCL, lower confidence limit; RR, relative risk; UCL, upper confidence limit. The trials included are as follows: 9 = Australasian Collaborative Trial of Magnesium Sulphate (ACTOMgSO_4_), 10 = PREterm brain protection by MAGnesium sulphate (PREMAG), 11 = Magnesium and Neurologic Endpoints Trial (MAGNET), and 12 = MAGnesium sulphate for Prevention of Eclampsia (MAGPIE).

^†^*p*-values for heterogeneity (The heterogeneity *p*-value for 1-stage analyses is from Wald chi-square tests for the interaction between treatment and subgroup in a generalising estimating equation [GEE] model).

#### F. Total dose received

The total dose of MgSO_4_ received varied by trial because of different treatment protocols; therefore, the effect of increased dose on primary outcomes was explored. Several different, prespecified combinations of dosage groupings were considered. No obvious linear trends were seen for any of the major outcomes when categorised by dose received, and no significant heterogeneity was observed (Table 16 and S4 Table).

**Table 16:** Treatment effects among the subgroups considered by dose actually received.

Outcome	Dose (g)	Included trials	MgSO_4_	Control	RR	LCL	UCL	*p*^†^
Death or CP	0 to <4	9,10,11,13	18/133 (13.5%)	20/141 (14.2%)	0.96	0.52	1.75	0.80
	4 to <14	9,10,11,13	178/963 (18.5%)	205/955 (21.5%)	0.86	0.72	1.04	–
	14 to <28	9,13	26/166 (15.7%)	42/191 (22.0%)	0.70	0.45	1.11	–
	28 to <42	13	75/598 (12.5%)	92/623 (14.8%)	0.86	0.64	1.15	–
	42 to <56	13	16/174 (9.20%)	18/186 (9.68%)	0.95	0.49	1.82	–
	56+	11,13	24/191 (12.6%)	15/152 (9.87%)	1.23	0.65	2.32	–
Death (at any time)	0 to <4	9,10,11,13	10/133 (7.52%)	15/141 (10.6%)	0.73	0.35	1.51	0.81
	4 to <14	9,10,11,13	126/963 (13.1%)	131/955 (13.7%)	0.97	0.76	1.23	–
	14 to <28	9,13	18/166 (10.8%)	24/191 (12.6%)	0.81	0.44	1.50	–
	28 to <42	13	51/598 (8.53%)	52/623 (8.35%)	1.02	0.70	1.48	–
	42 to <56	13	12/174 (6.90%)	14/186 (7.53%)	0.91	0.43	1.90	–
	56+	13	13/168 (7.74%)	7/148 (4.73%)	1.63	0.65	4.05	–
Cerebral palsy	0 to <4	9,10,11,13	8/119 (6.72%)	5/124 (4.03%)	1.71	0.50	5.80	0.44
	4	9,10,11	21/328 (6.40%)	32/283 (11.3%)	0.57	0.33	0.98	–
	4 to <14	9,13	31/503 (6.16%)	40/530 (7.55%)	0.82	0.52	1.28	–
	14 to <28	9,13	8/149 (5.37%)	18/176 (10.2%)	0.50	0.23	1.13	–
	28+	13	36/900 (4.00%)	52/911 (5.71%)	0.70	0.46	1.07	–
Death or major neurosensory disability	0 to <4	9,10,11,13	26/133 (19.5%)	28/141 (19.9%)	1.01	0.63	1.64	0.11
	4 to <14	9,10,11,13	244/963 (25.3%)	259/955 (27.1%)	0.98	0.83	1.14	–
	14 to <28	9,13	47/166 (28.3%)	76/191 (39.8%)	0.72	0.53	0.98	–
	28 to <42	13	204/598 (34.1%)	215/623 (34.5%)	1.00	0.85	1.18	–
	42 to <56	13	50/174 (28.7%)	44/186 (23.7%)	1.25	0.87	1.79	–
	56+	13	52/168 (31.0%)	33/148 (22.3%)	1.40	0.95	2.07	–

Abbreviations: CP, cerebral palsy; LCL, lower confidence limit; RR, relative risk; UCL, upper confidence limit. The trials included are as follows: 9 = Australasian Collaborative Trial of Magnesium Sulphate (ACTOMgSO_4_), 10 = PREterm brain protection by MAGnesium sulphate (PREMAG), 11 = Magnesium and Neurologic Endpoints Trial (MAGNET), 12 = MAGnesium sulphate for Prevention of Eclampsia (MAGPIE), and 13 = Beneficial Effects of Antenatal Magnesium Sulfate (BEAM). No significant interaction terms were observed for any endpoint when total dose was analysed as a continuous variable. Total dose itself was not significant in the model either.

^†^*p*-values for heterogeneity (the heterogeneity *p*-value for 1-stage analyses is from Wald chi-square tests for the interaction between treatment and subgroup in a generalising estimating equation [GEE] model).

#### G. Maintenance therapy received

There were no clear differences in treatment effects among the subgroups considered by whether maintenance therapy had been received or not (Table 17).

**Table 17:** Treatment effects among the subgroups considered by whether maintenance therapy was received or not.

Outcome	Maintenance	Included trials	MgSO_4_	Control	RR	LCL	UCL	*p*^†^
Death or CP	No^‡^	9,10	73/458 (15.9%)	90/417 (21.6%)	0.73	0.54	0.98	0.34
	Yes^§^	9,11	113/579 (19.5%)	129/559 (23.1%)	0.87	0.69	1.11	
Death (at any time)	No^‡^	9,10	48/458 (10.5%)	52/417 (12.5%)	0.83	0.56	1.23	0.99
	Yes^§^	9,11	80/579 (13.8%)	94/559 (16.8%)	0.83	0.62	1.11	
Cerebral palsy	No^‡^	9,10	25/401 (6.23%)	38/356 (10.7%)	0.59	0.36	0.97	0.17
	Yes^§^	9,11	33/481 (6.86%)	35/460 (7.61%)	0.94	0.59	1.49	
Death or major neurosensory disability	No^‡^	9,10	72/458 (15.7%)	81/417 (19.4%)	0.81	0.60	1.09	0.26
	Yes^§^	9,11	162/579 (28.0%)	166/559 (29.7%)	1.00	0.82	1.21	

Abbreviations: CP, cerebral palsy; LCL, lower confidence limit; RR, relative risk; UCL, upper confidence limit. The trials included are as follows: 9 = Australasian Collaborative Trial of Magnesium Sulphate (ACTOMgSO_4_), 10 = PREterm brain protection by MAGnesium sulphate (PREMAG), and 11 = Magnesium and Neurologic Endpoints Trial (MAGNET).

^†^*p*-values for heterogeneity for 1-stage analyses are from Wald chi-square tests for the interaction between treatment and subgroup in a generalising estimating equation (GEE) model.

^‡^At least some of the loading dose was received.

^§^The full loading dose was received, plus at least some of the maintenance dose

#### H. Whether repeat antenatal magnesium sulphate treatment was received

No trial was able to provide data to contribute to this planned subgroup analysis.

### Sensitivity analyses to account for missing data

Regardless of the methods used to account for missing data, no conclusions were altered compared with the main results reported above for any of the outcomes (S5 Table).

## Discussion

### Summary of evidence

The major finding from this IPD-MA is that for women at risk of imminent preterm birth antenatal, magnesium sulphate reduces the risk of their baby developing CP, with a NNT to benefit of 46 babies. The neuroprotective effect is confirmed when the primary reason for giving the antenatal magnesium sulphate is for neuroprotection of the fetus. Importantly, this benefit is not at the expense of an increase in mortality for the baby, and there appear to be no substantial short- or long-term complications for the mother or fetus from treatment with antenatal magnesium sulphate.

The results from this IPD-MA are consistent with the findings of the existing aggregate data meta-analysis in the Cochrane Library [14]. The new information that this IPD-MA has been able to clarify is that no particular subgroup of women and babies benefitted more or less from treatment among varying reasons for preterm birth, varying preterm gestational ages when treatment was started, or with varying dosage regimens, including whether maintenance therapy was given after a loading dose or not. Consequently, the information from the IPD-MA shows that there are no particular subgroups of women or their babies who might benefit more from treatment compared to others. The IPD-MA shows that antenatal magnesium sulphate reduces not only all CP, the majority of which is mild, but also moderate and severe combined and severe CP alone. Of note, the beneficial effect of magnesium sulphate on CP was not associated with fewer severe cerebral morbidities such as grade 3–4 intraventricular haemorrhage or cystic periventricular leucomalacia.

No severe adverse maternal events (including death or respiratory or cardiac arrest) were recorded for the 2 studies able to provide data for this IPD-MA. While reassuring, information about these rare maternal events should be routinely collected [26]. In combination with the known but less serious side effects of magnesium sulphate, such as flushing and tachycardia, the fact that maternal side effects are more likely to lead to stopping of a beneficial treatment than placebo and that severe maternal events are possible highlight the importance of giving magnesium sulphate under appropriate supervision [26,27].

The lack of an effect of magnesium sulphate on Apgar scores at 5 minutes, a need for active resuscitation at birth, or a need for ongoing assisted ventilation after birth indicates that magnesium sulphate, as used in these trials, does not have any substantial effect on depressing infant breathing after birth, either immediately or in the early hours and days of life, after which time the drug would usually have been excreted through the urinary system.

There were no substantial relationships between time from starting magnesium treatment until birth, the total dose received, or whether maintenance treatment was received and any of the major health outcomes. As these events all occur postrandomisation and therefore may be affected by the treatment given, interpretation of the results must be cautious. With maternal side effects increasing with higher total dose [26] and with concerns about maternal safety, at a clinical level it may be prudent to limit treatment to times close to birth and to minimise the dose of magnesium used to a 4-g bolus loading dose with or without a maintenance dose of 1 g/hour.

### Strengths and limitations

IPD-MA is recognised as the gold standard for systematic reviews [17]. One of the strengths of this study is that we have been able to include individual participant data from all of the known completed randomised clinical trials of magnesium sulphate for which developmental outcomes for the child have been reported.

We are aware of 2 ongoing placebo-controlled trials of antenatal magnesium sulphate for neuroprotection of the fetus that will report on childhood development: one in women at risk of preterm birth from 24 to <32 weeks’ gestation with a sample size of 500 [24] that is due to report in 2018 and the other where preterm birth is expected or planned between 30 to <34 weeks’ gestation, with a planned sample size of 1,676 babies and expected to report in 2021 [25]. Ultimately, data from these trials will be eligible to contribute to an update of this IPD-MA.

The limitations of our study are that not all trials had collected or could provide the data required for all of the prespecified analyses. Given that maternal and fetal event rates are low for some important clinical events, the power to find any overall or subgroup differences was limited. Lack of data from individual studies compounded the problem of low power for some of the analyses. Several of the planned analyses were for events that occurred after randomisation, including total dose received and the duration between start of treatment and birth. We recognise that such events are more subject to bias, but our intent was to identify subgroups of mothers who benefitted most and thus enable use of antenatal magnesium sulphate therapy to be better targeted.

## Conclusions

This IPD meta-analysis reaffirms that antenatal magnesium sulphate given prior to preterm birth is neuroprotective for CP and reduces the combined risk of fetal/infant death or CP, when the primary intent of magnesium sulphate treatment is fetal neuroprotection. Antenatal magnesium sulphate is a relatively inexpensive, easy to administer, effective treatment that can reduce the burden of death and CP facing babies born very preterm. Widespread adoption of the recommendations to use magnesium sulphate within several national clinical practice guidelines [15,16,28,29] and now within the recent WHO recommendations on interventions to improve preterm birth outcomes [3] could lead to significant global health benefits.

It is reassuring that antenatal magnesium sulphate does not appear to substantially depress the breathing efforts of babies either at birth or in the first hours of life. It is also reassuring that benefit is seen regardless of the reason for preterm birth and that antenatal magnesium sulphate has similar effects across a range of preterm gestational ages. As there is minimal variation in outcomes related to time to birth and with dosage, it would be prudent to restrict administration of antenatal magnesium sulphate for fetal neuroprotection to close to the expected or planned birth and to use 4 g, the smallest effective dose, with or without a 1 g/hour maintenance dose.

## Supporting information

S1 Table Contact information for each trial. (DOCX)

S2 Table Treatment effects among the subgroups of gestational age when treatment was first given—Other classification categories. (DOCX)

S3 Table Treatment effects by subgroups according to the time from the start of treatment until birth—Other classification categories. (DOCX)

S4 Table Treatment effects amongst the subgroups considered by dose actually received—Other classification categories. (DOCX)

S5 Table Individual study results for the secondary outcomes and sensitivity analyses. (DOCX)

S1 Text Preferred Reporting Items for Systematic Reviews and Meta-Analyses (PRISMA) checklist. (DOCX)

S2 Text AMICABLE (Antenatal magnesium sulphate individual participant data international collaboration: Assessing the benefits for babies using the best level of evidence) individual participant data meta-analysis (IPD-MA) study protocol. (PDF)

S3 Text Data items collected. (DOCX)

S4 Text Secondary outcomes. (DOCX)

## Abbreviations

ACTOMgSO_4_: Australasian Collaborative Trial of Magnesium Sulphate
AMICABLE: Antenatal magnesium individual participant data international collaboration: Assessing the benefits for babies using the best level of evidence
APH: antepartum haemorrhage
BEAM: Beneficial Effects of Antenatal Magnesium Sulfate
BPD: bronchopulmonary dysplasia
CENTRAL: Cochrane Central Register of Controlled Trials
CI: confidence interval
CP: cerebral palsy
GA: gestational age
GEE: generalising estimating equation
IPD-MA: individual participant data meta-analysis
IVH: intraventricular haemorrhage
LCL: lower confidence limit
MAGNET: Magnesium and Neurologic Endpoints Trial
MAGPIE: MAGnesium sulphate for Prevention of Eclampsia
MgSO_4_: magnesium sulphate
MI: multiple imputation
NEC: necrotising enterocolitis
NNT: number needed to treat
PDA: patent ductus arteriosus
PREMAG: PREterm brain protection by MAGnesium sulphate
PRISMA-IPD: Preferred Reporting Items for Systematic Review and Meta-analyses of Individual Participant Data
PROM: prelabour rupture of the membranes
PVL: periventricular leucomalacia
ROP: retinopathy of prematurity
RR: relative risk
UCL: upper confidence limit
WHO: World Health Organisation
