# Supplementary material for: Assessing the neuroprotective benefits for babies of antenatal magnesium sulphate: An individual participant data meta-analysis
Source: PLoS Med. 2017 Oct 4;14(10):e1002398. doi: 10.1371/journal.pmed.1002398 (PMC5627896; doi:10.1371/journal.pmed.1002398)
Supplement: S1 Table — (DOCX) [file pmed.1002398.s001.docx]

S1 Table. Contact person for the five studies.

| Name of Study | Study Contact Person | Contact Email |
| --- | --- | --- |
| ACTOMgSO_4_ | C. Crowther | c.crowther@auckland.ac.nz |
| PREMAG | S. Marret | Stephane.Marret@chu-rouen.fr |
| MAGNET | P. Pryde | ppryde57@gmail.com |
| MAGPIE | L. Duley | Lelia.Duley@nottingham.ac.uk |
| BEAM | D. Rouse | Dwight_Rouse@brown.edu |
