# Supplementary material for: Assessing the neuroprotective benefits for babies of antenatal magnesium sulphate: An individual participant data meta-analysis
Source: PLoS Med. 2017 Oct 4;14(10):e1002398. doi: 10.1371/journal.pmed.1002398 (PMC5627896; doi:10.1371/journal.pmed.1002398)
Supplement: S2 Table — (DOCX) [file pmed.1002398.s002.docx]

S2 Table. Treatment effects among the subgroups considered by gestational age when treatment first given - other classification categories

| **Outcome** | **GA (weeks)** | **Included Trials** | **MgSO4** | **Control** | **RR** | **LCL** | **UCL** | **P†** |
| --- | --- | --- | --- | --- | --- | --- | --- | --- |
| Death or CP | <28 | 9,10,11,12,13 | 313/1039 (30.1%) | 355/1100 (32.3%) | 0.97 | 0.86 | 1.08 | 0.67 |
|  | 28+ | 9,10,11,12,13 | 229/2006 (11.4%) | 222/1983 (11.2%) | 1.01 | 0.85 | 1.21 | . |
|  | <30 | 9,10,11,12,13 | 411/1771 (23.2%) | 453/1806 (25.1%) | 0.96 | 0.86 | 1.07 | 0.49 |
|  | 30+ | 10,11,12,13 | 131/1274 (10.3%) | 124/1277 (9.7%) | 1.05 | 0.84 | 1.32 | . |
| Death (at any | <28 | 9,10,11,12,13 | 246/1039 (23.7%) | 258/1100 (23.5%) | 1.04 | 0.92 | 1.18 | 0.86 |
| time) | 28+ | 9,10,11,12,13 | 190/2006 (9.5%) | 163/1983 (8.2%) | 1.14 | 0.93 | 1.38 | . |
|  | <30 | 9,10,11,12,13 | 317/1771 (17.9%) | 318/1806 (17.6%) | 1.04 | 0.92 | 1.18 | 0.57 |
|  | 30+ | 10,11,12,13 | 119/1274 (9.3%) | 103/1277 (8.1%) | 1.14 | 0.89 | 1.46 | . |
| Cerebral | <28 | 9,10,11,13 | 67/837 (8.0%) | 98/876 (11.2%) | 0.69 | 0.52 | 0.93 | 0.86 |
| Palsy | 28+ | 9,10,11,12,13 | 39/1432 (2.7%) | 59/1441 (4.1%) | 0.67 | 0.44 | 1.00 | . |
|  | <30 | 9,10,11,12,13 | 94/1478 (6.4%) | 136/1500 (9.1%) | 0.69 | 0.54 | 0.89 | 0.71 |
|  | 30+ | 10,12,13 | 12/763 (1.6%) | 21/792 (2.7%) | 0.62 | 0.30 | 1.26 | . |
| Death or | <28 | 9,10,11,12,13 | 459/1039 (44.2%) | 492/1100 (44.7%) | 1.02 | 0.93 | 1.12 | 0.54 |
| major neurosensory disability | 28+ | 9,10,11,12,13 | 380/2006 (18.9%) | 356/1983 (18.0%) | 1.06 | 0.93 | 1.22 | . |
|  | <30 | 9,10,11,12,13 | 638/1771 (36.0%) | 653/1806 (36.2%) | 1.02 | 0.94 | 1.11 | 0.72 |
|  | 30+ | 10,11,12,13 | 201/1274 (15.8%) | 195/1277 (15.3%) | 1.06 | 0.88 | 1.27 |  |

Trials included: 9=ACTOMgSO_4_, 10=PREMAG, 11=MAGNET, 12=MAGPIE, 13=BEAM.

GA=gestational age; RR=Relative Risk; LC =95% Lower confidence limit; UCL=95% Upper confidence limit; CP=Cerebral palsy; MI=multiple imputation;

† Heterogeneity p values for one-stage analyses are from Wald chi-square tests for the interaction between treatment and subgroup in a GEE model.
