# Supplementary material for: Assessing the neuroprotective benefits for babies of antenatal magnesium sulphate: An individual participant data meta-analysis
Source: PLoS Med. 2017 Oct 4;14(10):e1002398. doi: 10.1371/journal.pmed.1002398 (PMC5627896; doi:10.1371/journal.pmed.1002398)
Supplement: S3 Table — (DOCX) [file pmed.1002398.s003.docx]

S3 Table. Treatment effects among the subgroups considered by time from start of treatment to birth - other classification categories

| **Outcome** | **Time (hours)** | **Included Trials** | **MgSO4** | **Control** | **RR** | **LCL** | **UCL** | **P†** |
| --- | --- | --- | --- | --- | --- | --- | --- | --- |
| Cerebral | 0 to <4 | 9,10,11 | 40/558 (7.2%) | 53/551 (9.6%) | 0.75 | 0.50 | 1.12 | 0.74 |
| Palsy | 4 to <8 | 9,10,12 | 9/131 (6.9%) | 7/126 (5.6%) | 1.08 | 0.43 | 2.74 | . |
|  | >8 | 9,10,11,12 | 13/378 (3.4%) | 14/318 (4.4%) | 0.81 | 0.37 | 1.78 | . |

Trials included: 9=ACTOMgSO_4_, 10=PREMAG, 11=MAGNET, 12=MAGPIE

RR=Relative Risk; LCL = 95% Lower confidence limit; UCL = 95% Upper confidence limit; CP=Cerebral palsy;

† Heterogeneity p values for one-stage analyses are from Wald chi-square tests for the interaction between treatment and subgroup in a GEE model.
