# Supplementary material for: Assessing the neuroprotective benefits for babies of antenatal magnesium sulphate: An individual participant data meta-analysis
Source: PLoS Med. 2017 Oct 4;14(10):e1002398. doi: 10.1371/journal.pmed.1002398 (PMC5627896; doi:10.1371/journal.pmed.1002398)
Supplement: S4 Table — (DOCX) [file pmed.1002398.s004.docx]

S4 Table. Treatment effects among the subgroups considered by dose actually received - other classification categories

| **Outcome** | **Dose (g)** | **Included Trials** | **MgSO4** | **Control** | **RR** | **LCL** | **UCL** | **P†** |
| --- | --- | --- | --- | --- | --- | --- | --- | --- |
| Death or CP | 0 to <4 | 9,10,11,13 | 18/133 (13.5%) | 20/141 (14.2%) | 0.96 | 0.52 | 1.75 | 0.77 |
|  | 4 to <14 | 9,10,11,13 | 178/963 (18.5%) | 205/955 (21.5%) | 0.86 | 0.72 | 1.04 | . |
|  | 14 to <28 | 9,13 | 26/166 (15.7%) | 42/191 (22.0%) | 0.70 | 0.45 | 1.11 | . |
|  | 28+ | 11,13 | 117/983 (11.9%) | 125/965 (13.0%) | 0.92 | 0.72 | 1.17 | . |
|  |  |  |  |  |  |  |  |  |
|  | 0 to <4 | 9,10,11,13 | 18/133 (13.5%) | 20/141 (14.2%) | 0.96 | 0.52 | 1.75 | 0.80 |
|  | 4 | 9,10,11 | 65/382 (17.0%) | 71/333 (21.3%) | 0.79 | 0.57 | 1.08 | . |
|  | >4 | 9,11,13 | 257/1737 (14.8%) | 299/1778 (16.8%) | 0.89 | 0.76 | 1.04 | . |
|  | 0 to <4 | 9,10,11,13 | 18/133 (13.5%) | 20/141 (14.2%) | 0.96 | 0.52 | 1.75 | 0.82 |
|  | 4 | 9,10,11 | 65/382 (17.0%) | 71/333 (21.3%) | 0.79 | 0.57 | 1.08 | . |
|  | 4 to <14 | 9,13 | 112/576 (19.4%) | 132/618 (21.4%) | 0.91 | 0.72 | 1.15 | . |
|  | 14 to <28 | 9,11,13 | 27/173 (15.6%) | 42/193 (21.8%) | 0.72 | 0.46 | 1.13 | . |
|  | 28+ | 11,13 | 117/983 (11.9%) | 125/965 (13.0%) | 0.92 | 0.72 | 1.17 | . |
| Death (at any | 0 to <4 | 9,10,11,13 | 10/133 (7.52%) | 15/141 (10.6%) | 0.73 | 0.35 | 1.51 | 0.74 |
| time) | 4 to <14 | 9,10,11,13 | 126/963 (13.1%) | 131/955 (13.7%) | 0.97 | 0.76 | 1.23 | . |
|  | 14 to <28 | 9,13 | 18/166 (10.8%) | 24/191 (12.6%) | 0.81 | 0.44 | 1.50 | . |
|  | 28+ | 13 | 76/940 (8.1%) | 73/957 (7.6%) | 1.06 | 0.77 | 1.45 | . |
|  |  |  |  |  |  |  |  |  |
|  | 0 to <4 | 9,10,11,13 | 10/133 (7.52%) | 15/141 (10.6%) | 0.73 | 0.35 | 1.51 | 0.68 |
|  | 4 | 9,10,11 | 44/382 (11.5%) | 39/333 (11.7%) | 0.98 | 0.64 | 1.51 | . |
|  | >4 | 9,11,13 | 182/1737 (10.5%) | 189/1778 (10.6%) | 0.98 | 0.80 | 1.20 | . |
|  | 0 to <4 | 9,10,11,13 | 10/133 (7.5%) | 15/141 (10.6%) | 0.73 | 0.35 | 1.51 | 0.87 |
|  | 4 | 9,10,11 | 44/382 (11.5%) | 39/333 (11.7%) | 0.98 | 0.64 | 1.51 | . |
|  | 4 to <14 | 9,11,13 | 82/581 (14.1%) | 92/620 (14.8%) | 0.95 | 0.71 | 1.27 | . |
|  | 14 to <28 | 9,11,13 | 19/173 (11.0%) | 24/193 (12.4%) | 0.84 | 0.46 | 1.52 | . |
|  | 28+ | 13 | 76/940 (8.1%) | 73/957 (7.6%) | 1.06 | 0.77 | 1.45 | . |
| Cerebral | 0 to <4 | 9,10,11,13 | 8/119 (6.7%) | 5/124 (4.0%) | 1.71 | 0.50 | 5.80 | 0.26 |
| Palsy | 4 to <14 | 9,10,11,13 | 52/834 (6.2%) | 74/817 (9.1%) | 0.68 | 0.48 | 0.96 | . |
|  | 14+ | 9,11,13 | 44/1082 (4.1%) | 71/1095 (6.5%) | 0.63 | 0.44 | 0.92 | . |
|  |  |  |  |  |  |  |  |  |
|  | 0 to <4 | 9,10,11,13 | 8/119 (6.7%) | 5/124 (4.0%) | 1.71 | 0.50 | 5.80 | 0.26 |
|  | 4 | 9,10,11 | 21/328 (6.4%) | 32/283 (11.3%) | 0.57 | 0.33 | 0.98 | . |
|  | >4 | 9,11,13 | 75/1588 (4.7%) | 111/1627 (6.8%) | 0.70 | 0.53 | 0.93 | . |
| Death or | 0 to <4 | 9,10,11,13 | 26/133 (19.5%) | 28/141 (19.9%) | 1.01 | 0.63 | 1.64 | 0.11 |
| major | 4 to <14 | 9,10,11,13 | 244/963 (25.3%) | 259/955 (27.1%) | 0.98 | 0.83 | 1.14 | . |
| neurosensory | 14 to <28 | 9,13 | 47/166 (28.3%) | 76/191 (39.8%) | 0.72 | 0.53 | 0.98 | . |
| disability | 28+ | 13 | 306/940 (32.6%) | 292/957 (30.5%) | 1.08 | 0.94 | 1.24 | . |
|  |  |  |  |  |  |  |  |  |
|  | 0 to <4 | 19,10,11,13 | 26/133 (19.5%) | 28/141 (19.9%) | 1.01 | 0.63 | 1.64 | 0.36 |
|  | 4 | 1,2,3 | 58/382 (15.2%) | 64/333 (19.2%) | 0.79 | 0.56 | 1.10 | . |
|  | >4 | 1,3,5 | 546/1737 (31.4%) | 563/1778 (31.7%) | 1.02 | 0.92 | 1.12 | . |
|  |  |  |  |  |  |  |  |  |
|  | 0 to <4 | 9,10,11,13 | 26/133 (19.5%) | 28/141 (19.9%) | 1.01 | 0.63 | 1.64 | 0.10 |
|  | 4 | 1,2,3 | 58/382 (15.2%) | 64/333 (19.2%) | 0.79 | 0.56 | 1.10 | . |
|  | 4 to <14 | 1,3,5 | 186/581 (32.0%) | 195/620 (31.5%) | 1.03 | 0.87 | 1.23 | . |
|  | 14 to <28 | 1,3,5 | 48/173 (27.7%) | 76/193 (39.4%) | 0.73 | 0.54 | 0.99 | . |
|  | 28+ | 13 | 306/940 (32.6%) | 292/957 (30.5%) | 1.08 | 0.94 | 1.24 | . |

Trials included: 9=ACTOMgSO_4_, 10=PREMAG, 11=MAGNET, 13=BEAM.

RR=Relative Risk; LCL = 95% Lower confidence limit; UCL = 95% Upper confidence limit; CP=Cerebral palsy;

†Heterogeneity p values for one-stage analyses are from Wald chi-square tests for the interaction between treatment and subgroup in a GEE model.
