# Supplementary material for: Assessing the neuroprotective benefits for babies of antenatal magnesium sulphate: An individual participant data meta-analysis
Source: PLoS Med. 2017 Oct 4;14(10):e1002398. doi: 10.1371/journal.pmed.1002398 (PMC5627896; doi:10.1371/journal.pmed.1002398)
Supplement: S5 Table — (DOCX) [file pmed.1002398.s005.docx]

S5 Table. Individual study results for the secondary outcomes and sensitivity analyses.

[Table 1 Death or Cerebral Palsy (CP as defined by trialist) 3](#_Toc489375072)

[Table 2 Infant death (fetal, neonatal, later death) at any time 5](#_Toc489375073)

[Table 3 Infant death at any time (fetal, neonatal or later) (Fetal neuroprotective intent only trials) 6](#_Toc489375074)

[Table 4 Cerebral Palsy (as defined by trialist) 6](#_Toc489375075)

[Table 5 Cerebral palsy (Fetal neuroprotective intent only trials) 7](#_Toc489375076)

[Table 6 At least moderate CP and at least severe CP 7](#_Toc489375077)

[Table 7 Apgar score at 5 mins < 7 8](#_Toc489375078)

[Table 8 Active resuscitation at birth 8](#_Toc489375079)

[Table 9 Respiratory Distress Syndrome 9](#_Toc489375080)

[Table 10 Use of ongoing respiratory support 9](#_Toc489375081)

[Table 11 Chronic Lung Disease/bronchopulmonary dysplasia (as defined by trialist) 10](#_Toc489375082)

[Table 12 Neonatal convulsions 10](#_Toc489375083)

[Table 13 Intraventricular haemorrhage (any) 11](#_Toc489375084)

[Table 14 Severe intraventricular haemorrhage (Grade 3 or 4) 11](#_Toc489375085)

[Table 15 Cystic periventricular leukomalacia 12](#_Toc489375086)

[Table 16 Post haemorrhagic hydrocephaly or ventriculomegaly 12](#_Toc489375087)

[Table 17 Proven neonatal systemic infection 13](#_Toc489375088)

[Table 18 Necrotising enterocolitis 13](#_Toc489375089)

[Table 19 Patent ductus arteriosus requiring treatment 14](#_Toc489375090)

[Table 20 Any retinopathy of prematurity 14](#_Toc489375091)

[Table 21 Severe neonatal adverse outcome 15](#_Toc489375092)

[Table 22 Developmental Delay or Intellectual Impairment at follow up 15](#_Toc489375093)

[Table 23 At least Moderate or at Least Severe Developmental Delay 16](#_Toc489375094)

[Table 24 Blindness 16](#_Toc489375095)

[Table 25 Deafness 17](#_Toc489375096)

[Table 26 Gross motor dysfunction (any) 17](#_Toc489375097)

[Table 27 Gross motor dysfunction (moderate or severe) 18](#_Toc489375098)

[Table 28 Neurosensory disability 18](#_Toc489375099)

[Table 29 Major neurosensory disability 19](#_Toc489375100)

[Table 30 Psychomotor dysfunction 19](#_Toc489375101)

[Table 31 Death or major neurosensory disability 20](#_Toc489375102)

[Table 32 Death or any neurosensory disability 20](#_Toc489375103)

[Table 33 Death or moderate-severe gross motor dysfunction 21](#_Toc489375104)

[Table 34 Length of maternal postnatal stay 21](#_Toc489375105)

[Table 35 Gestational age at birth (weeks) 22](#_Toc489375106)

[Table 36 Growth measurements at birth 22](#_Toc489375107)

[Table 37 Growth measurements at follow up 24](#_Toc489375108)

[Table 38 Adverse event enough to stop treatment 25](#_Toc489375109)

[Table 39 Intrapartum fever requiring antibiotics 26](#_Toc489375110)

[Table 40 Postpartum haemorrhage (>=500ml) 26](#_Toc489375111)

[Table 41 Mode of Birth (Caesarean) 26](#_Toc489375112)

[Table 42 Chorioamnionitis during labour 27](#_Toc489375113)

## Sensitivity analysis of primary outcomes

Table 1 Death or Cerebral Palsy (CP as defined by trialist)

| Death Or CP | *Trial* | *MgSO4* | *Control* | *RR* | *LCL* | *UCL* | *P: hetero geneity†* |
| --- | --- | --- | --- | --- | --- | --- | --- |
| Available Data | Crowther | 123/ 629 (19.6%) | 150/ 626 (24.0%) | 0.83 | 0.67 | 1.04 | . |
| (participants included if either Death or CP outcome known) | Marret | 56/ 353 (15.9%) | 68/ 338 (20.1%) | 0.76 | 0.54 | 1.07 | . |
|  | Mittendorf | 13/ 86 (15.1%) | 4/ 80 (5.00%) | 2.80 | 0.94 | 8.34 | . |
|  | Magpie | 202/ 790 (25.6%) | 182/ 785 (23.2%) | 1.07 | 0.90 | 1.28 | . |
|  | Rouse | 148/1188 (12.5%) | 173/1256 (13.8%) | 0.91 | 0.74 | 1.12 | . |
|  | **OVERALL** | 542/3046 (17.8%) | 577/3085 (18.7%) | 0.94 | 0.85 | 1.05 | 0.0665 |
| Multiple Imputations* for missing CP | Crowther | 123/ 629 (19.6%) | 150/ 626 (24.0%) | 0.83 | 0.67 | 1.04 | . |
|  | Marret | 56/ 353 (15.9%) | 68/ 338 (20.1%) | 0.76 | 0.54 | 1.07 | . |
|  | *Mittendorf** | 14/ 86 (16.3%) | 5/ 80 (6.25%) | 2.38 | 0.81 | 7.02 | . |
|  | *Magpie** | 209/ 790 (26.5%) | 190/ 785 (24.2%) | 1.06 | 0.89 | 1.27 |  |
|  | Rouse | 148/1188 (12.5%) | 173/1256 (13.8%) | 0.91 | 0.74 | 1.12 |  |
| **IPD + MI (two stage)** | **OVERALL** | 550/3046 (18.1%) | 586/3085 (19.0%) | 0.94 | 0.87 | 1.01 | 0.1166 |
| Method: Delete Trial | Crowther | 123/ 629 (19.6%) | 150/ 626 (24.0%) | 0.83 | 0.67 | 1.04 | . |
|  | Marret | 56/ 353 (15.9%) | 68/ 338 (20.1%) | 0.76 | 0.54 | 1.07 | . |
|  | Mittendorf | 13/ 86 (15.1%) | 4/ 80 (5.00%) | 2.80 | 0.94 | 8.34 | . |
|  | Rouse | 148/1188 (12.5%) | 173/1256 (13.8%) | 0.91 | 0.74 | 1.12 | . |
|  | **OVERALL** | 340/2256 (15.1%) | 395/2300 (17.2%) | 0.87 | 0.76 | 1.00 | 0.1564 |
| Method: Impute ‘No CP’ for missing CP | Crowther | 123/ 629 (19.6%) | 150/ 626 (24.0%) | 0.83 | 0.67 | 1.04 | . |
|  | Marret | 56/ 353 (15.9%) | 68/ 338 (20.1%) | 0.76 | 0.54 | 1.07 | . |
|  | Mittendorf | 13/ 86 (15.1%) | 4/ 80 (5.00%) | 2.80 | 0.94 | 8.34 | . |
|  | Magpie | 202/ 790 (25.6%) | 182/ 785 (23.2%) | 1.07 | 0.90 | 1.28 | . |
|  | Rouse | 148/1188 (12.5%) | 173/1256 (13.8%) | 0.91 | 0.74 | 1.12 | . |
|  | **OVERALL** | 542/3046 (17.8%) | 577/3085 (18.7%) | 0.94 | 0.85 | 1.05 | 0.0665 |

W=inverse variance; RR=Relative Risk; LCL = 95% Lower confidence limit; UCL = 95% Upper confidence limit; CP=Cerebral palsy; MI=multiple imputation; IPD=Individual patient data;

*estimates are from multiple imputations analyses are shown in italics. 100 MI datasets were imputed using Stata version 11.2 ‘ice’ function. Counts and percentages within each treatment group for MI analyses represent average percentages in imputed datasets. Estimates and standard errors for imputed analyses are from multiple imputations GEE models adjusting for correlation between multiple births. Overall RR estimates were calculated using a two-stage aggregate meta-analysis approach with inverse variance weighting.

† Heterogeneity p values for one-stage analyses are from Wald chi-square tests for the interaction between treatment and trial in a GEE model.

The “Available Data” and “Impute ‘No’ for missing CP” methods are the same due to the fact that this is a composite endpoint of both Death and CP. All participants had death data and those with missing CP were imputed as ‘No CP’

##

## Secondary Endpoints

Table 2 Infant death (fetal, neonatal, later death) at any time

| *DESCRIPTION* | *Trial* | *MgSO4* | *Control* | *RR* | *LCL* | *UCL* | *P: Hetero geneity†* |
| --- | --- | --- | --- | --- | --- | --- | --- |
| Available Data | Crowther | 87/ 629 (13.8%) | 108/ 626 (17.3%) | 0.81 | 0.62 | 1.07 | . |
|  | Marret | 34/ 353 (9.63%) | 38/ 338 (11.2%) | 0.83 | 0.52 | 1.32 | . |
|  | Mittendorf | 10/ 86 (11.6%) | 1/ 80 (1.25%) | 8.11 | 1.05 | 62.69 | . |
|  | Magpie | 200/ 790 (25.3%) | 177/ 785 (22.5%) | 1.09 | 0.91 | 1.30 | . |
|  | Rouse‡ | 105/1188 (8.84%) | 97/1256 (7.72%) | 1.15 | 0.88 | 1.51 | . |
|  | **OVERALL** | 436/3046 (14.3%) | 421/3085 (13.6%) | 1.03 | 0.91 | 1.17 | 0.0687 |

W=inverse variance; RR=Relative Risk; LCL = 95% Lower confidence limit; UCL = 95% Upper confidence limit; IPD=Individual patient data;

All trials had data for deaths therefore there are no additional analyses to account for missingness. No imputations were undertaken for this endpoint.

‡The Rouse trial only included deaths in the first 12 months.

† Heterogeneity p values for one-stage analyses are from Wald chi-square tests for the interaction between treatment and trial in a GEE model.

Table 3 Infant death at any time (fetal, neonatal or later) (Fetal neuroprotective intent only trials)

| *DESCRIPTION* | *Trial* | *MgSO4* | *Control* | *RR* | *LCL* | *UCL* | *P: Hetero geneity*† | *W (%)* |
| --- | --- | --- | --- | --- | --- | --- | --- | --- |
| Available Data | Crowther | 87/ 629 (13.8%) | 108/ 626 (17.3%) | 0.81 | 0.62 | 1.07 | . | 42 |
|  | Marret | 34/ 353 (9.63%) | 38/ 338 (11.2%) | 0.83 | 0.52 | 1.32 | . | 15 |
|  | Mittendorf | 2/ 28 (7.14%) | 1/ 30 (3.33%) | 2.14 | 0.20 | 22.42 | . | 1 |
|  | Rouse‡ | 105/1188 (8.84%) | 97/1256 (7.72%) | 1.15 | 0.88 | 1.51 | . | 43 |
|  | **OVERALL** | 228/2198 (10.4%) | 244/2250 (10.8%) | 0.95 | 0.80 | 1.13 | 0.2668 | . |

W=inverse variance; RR=Relative Risk; LCL = 95% Lower confidence limit; UCL = 95% Upper confidence limit; CP=Cerebral palsy; IPD=Individual patient data;

† Heterogeneity p values for one-stage analyses are from Wald chi-square tests for the interaction between treatment and trial in a GEE model.

‡The Rouse trial only included deaths in the first 12 months.

Table 4 Cerebral Palsy (as defined by trialist)

| *DESCRIPTION* | *Trial* | *MgSO4* | *Control* | *RR* | *LCL* | *UCL* | *P: hetero geneity†* |
| --- | --- | --- | --- | --- | --- | --- | --- |
| Available Data | Crowther | 36/ 533 (6.75%) | 42/ 513 (8.19%) | 0.83 | 0.54 | 1.28 | . |
|  | Marret | 22/ 313 (7.03%) | 30/ 293 (10.2%) | 0.68 | 0.40 | 1.17 | . |
|  | Mittendorf | 3/ 60 (5.00%) | 3/ 62 (4.84%) | 1.03 | 0.22 | 4.94 | . |
|  | Magpie | 2/ 236 (0.85%) | 5/ 255 (1.96%) | 0.43 | 0.08 | 2.21 | . |
|  | Rouse | 43/1133 (3.80%) | 77/1203 (6.40%) | 0.59 | 0.41 | 0.86 | . |
|  | **OVERALL** | 106/2275 (4.66%) | 157/2326 (6.75%) | 0.68 | 0.54 | 0.87 | 0.7380 |

W=inverse variance; RR=Relative Risk; LCL = 95% Lower confidence limit; UCL = 95% Upper confidence limit; CP=Cerebral palsy; MI=multiple imputation; IPD=Individual patient data;

† Heterogeneity p values for one-stage analyses are from Wald chi-square tests for the interaction between treatment and trial in a GEE model.

Table 5 Cerebral palsy (Fetal neuroprotective intent only trials)

| *Description* | *Trial* | *MgSO4* | *Control* | *RR* | *LCL* | *UCL* | *P: hetero geneity†* |
| --- | --- | --- | --- | --- | --- | --- | --- |
| Cerebral palsy (available data) | Crowther | 36/ 533 (6.75%) | 42/ 513 (8.19%) | 0.83 | 0.54 | 1.28 | . |
|  | Marret | 22/ 313 (7.03%) | 30/ 293 (10.2%) | 0.68 | 0.40 | 1.17 | . |
|  | Mittendorf | 3/ 23 (13.0%) | 0/ 23 (0.00%) |  |  |  |  |
|  | Rouse | 43/1133 (3.80%) | 77/1203 (6.40%) | 0.59 | 0.41 | 0.86 | . |
|  | **OVERALL** | 101/1979 (5.10%) | 149/2009 (7.42%) | 0.68 | 0.53 | 0.87 | 0.4918 |

W=inverse variance; RR=Relative Risk; LCL = 95% Lower confidence limit; UCL = 95% Upper confidence limit; IPD=Individual patient data;

† Heterogeneity p values for one-stage analyses are from Wald chi-square tests for the interaction between treatment and trial in a GEE model.

Table 6 At least moderate CP and at least severe CP

| *DESCRIPTION* | *Trial* | *MgSO4* | *Control* | *RR* | *LCL* | *UCL* | *P: Hetero geneity* |
| --- | --- | --- | --- | --- | --- | --- | --- |
| At least moderate CP (Available data) | Crowther | 15/ 533 (2.81%) | 21/ 513 (4.09%) | 0.69 | 0.36 | 1.31 | . |
|  | Marret | 10/ 313 (3.19%) | 13/ 293 (4.44%) | 0.72 | 0.32 | 1.63 | . |
|  | Mittendorf | 1/ 3 (33.3%) | 1/ 4 (25.0%) | 1.33 | 0.13 | 13.74 | . |
|  | Magpie | 2/ 236 (0.85%) | 5/ 255 (1.96%) | 0.43 | 0.08 | 2.21 | . |
|  | Rouse | 19/1131 (1.68%) | 36/1200 (3.00%) | 0.56 | 0.32 | 0.97 | . |
|  | **OVERALL** | 47/2216 (2.12%) | 76/2265 (3.36%) | 0.63 | 0.44 | 0.90 | 0.9185 |
|  |  |  |  | . | . | . | . |
| At least severe CP (Available data) | Crowther | 3/ 533 (0.56%) | 6/ 513 (1.17%) | 0.48 | 0.12 | 1.91 | . |
|  | Marret | 8/ 313 (2.56%) | 7/ 293 (2.39%) | 1.07 | 0.39 | 2.92 | . |
|  | Magpie | 2/ 236 (0.85%) | 5/ 255 (1.96%) | 0.43 | 0.08 | 2.21 | . |
|  | Rouse | 5/1131 (0.44%) | 16/1200 (1.33%) | 0.33 | 0.12 | 0.90 | . |
|  | **OVERALL** | 18/2213 (0.81%) | 34/2261 (1.50%) | 0.54 | 0.30 | 0.94 | 0.4262 |

Table 7 Apgar score at 5 mins < 7

| *DESCRIPTION* | *Trial* | *MgSO4* | *Control* | *RR* | *LCL* | *UCL* | *P: Hetero geneity†* |
| --- | --- | --- | --- | --- | --- | --- | --- |
| Apgar score at 5 mins < 7 (Available data) | Crowther | 103/ 628 (16.4%) | 102/ 626 (16.3%) | 1.01 | 0.78 | 1.30 | . |
|  | Marret | 45/ 351 (12.8%) | 31/ 336 (9.23%) | 1.44 | 0.92 | 2.27 | . |
|  | Mittendorf | 1/ 72 (1.39%) | 4/ 75 (5.33%) | 0.26 | 0.03 | 2.28 | . |
|  | Magpie | 48/ 676 (7.10%) | 46/ 676 (6.80%) | 1.04 | 0.71 | 1.54 | . |
|  | Rouse | 217/1176 (18.5%) | 237/1248 (19.0%) | 0.95 | 0.80 | 1.13 | . |
|  | **OVERALL** | 414/2903 (14.3%) | 420/2961 (14.2%) | 1.01 | 0.89 | 1.14 | 0.3831 |

W=inverse variance; RR=Relative Risk; LCL = 95% Lower confidence limit; UCL = 95% Upper confidence limit; IPD=Individual patient data;

† Heterogeneity p values for one-stage analyses are from Wald chi-square tests for the interaction between treatment and trial in a GEE model.

Table 8 Active resuscitation at birth

| *DESCRIPTION* | *Trial* | *MgSO4* | *Control* | *RR* | *LCL* | *UCL* | *P: Hetero geneity†* |
| --- | --- | --- | --- | --- | --- | --- | --- |
| Active resuscitation at birth (available data) | Crowther | 595/ 629 (94.6%) | 595/ 626 (95.0%) | 1.00 | 0.97 | 1.02 | . |
|  | Marret | 117/ 350 (33.4%) | 103/ 335 (30.7%) | 1.05 | 0.83 | 1.32 | . |
|  | Magpie | 52/ 680 (7.65%) | 59/ 678 (8.70%) | 0.88 | 0.62 | 1.27 | . |
|  | Rouse | 454/1177 (38.6%) | 527/1252 (42.1%) | 0.93 | 0.84 | 1.02 | . |
|  | **OVERALL** | 1218/2836 (42.9%) | 1284/2891 (44.4%) | 0.99 | 0.96 | 1.02 | 0.4574 |

W=inverse variance; RR=Relative Risk; LCL = 95% Lower confidence limit; UCL = 95% Upper confidence limit; IPD=Individual patient data;

† Heterogeneity p values for one-stage analyses are from Wald chi-square tests for the interaction between treatment and trial in a GEE model.

Table 9 Respiratory Distress Syndrome

| *Description* | *Trial* | *MgSO4* | *Control* | *RR* | *LCL* | *UCL* | *P: Hetero geneity†* |
| --- | --- | --- | --- | --- | --- | --- | --- |
| Respiratory distress syndrome (available data) | Crowther | 540/ 629 (85.9%) | 526/ 626 (84.0%) | 1.02 | 0.97 | 1.08 | . |
|  | Marret | 145/ 345 (42.0%) | 123/ 325 (37.8%) | 1.08 | 0.89 | 1.32 | . |
|  | Mittendorf | 18/ 70 (25.7%) | 20/ 73 (27.4%) | 0.97 | 0.56 | 1.68 | . |
|  | Rouse | 585/1171 (50.0%) | 656/1244 (52.7%) | 0.96 | 0.88 | 1.04 | . |
|  | **OVERALL** | 1288/2215 (58.1%) | 1325/2268 (58.4%) | 1.01 | 0.97 | 1.05 | 0.4618 |

Table 10 Use of ongoing respiratory support

| *Description* | *Trial* | *MgSO4* | *Control* | *RR* | *LCL* | *UCL* | *P: Hetero geneity†* |
| --- | --- | --- | --- | --- | --- | --- | --- |
| Use of respiratory support (available data) | Crowther | 577/ 629 (91.7%) | 562/ 626 (89.8%) | 1.02 | 0.98 | 1.06 | . |
|  | Marret | 192/ 345 (55.7%) | 176/ 325 (54.2%) | 1.00 | 0.87 | 1.16 | . |
|  | Mittendorf | 23/ 72 (31.9%) | 18/ 74 (24.3%) | 1.35 | 0.79 | 2.31 | . |
|  | Magpie | 139/ 450 (30.9%) | 147/ 442 (33.3%) | 0.93 | 0.77 | 1.13 | . |
|  | Rouse | 613/1171 (52.3%) | 709/1243 (57.0%) | 0.92 | 0.85 | 0.99 | . |
|  | **OVERALL** | 1544/2667 (57.9%) | 1612/2710 (59.5%) | 1.00 | 0.97 | 1.03 | 0.0993 |

W=inverse variance; RR=Relative Risk; LCL = 95% Lower confidence limit; UCL = 95% Upper confidence limit; IPD=Individual patient data; † Heterogeneity p values for one-stage analyses are from Wald chi-square tests for the interaction between treatment and trial in a GEE model.

Table 11 Chronic Lung Disease/bronchopulmonary dysplasia (as defined by trialist)

| *Description* | *Trial* | *MgSO4* | *Control* | *RR* | *LCL* | *UCL* | *P: Hetero geneity†* |
| --- | --- | --- | --- | --- | --- | --- | --- |
| Chronic lung disease (available data) | Crowther | 192/ 629 (30.5%) | 164/ 626 (26.2%) | 1.16 | 0.97 | 1.39 | . |
|  | Marret | 28/ 351 (7.98%) | 31/ 329 (9.42%) | 0.83 | 0.50 | 1.38 | . |
|  | Mittendorf | 9/ 72 (12.5%) | 7/ 74 (9.46%) | 1.32 | 0.52 | 3.34 | . |
|  | Rouse | 213/1171 (18.2%) | 218/1244 (17.5%) | 1.03 | 0.86 | 1.23 | . |
|  | **OVERALL** | 442/2223 (19.9%) | 420/2273 (18.5%) | 1.08 | 0.96 | 1.22 | 0.5625 |

W=inverse variance; RR=Relative Risk; LCL = 95% Lower confidence limit; UCL = 95% Upper confidence limit; IPD=Individual patient data;

† Heterogeneity p values for one-stage analyses are from Wald chi-square tests for the interaction between treatment and trial in a GEE model.

Table 12 Neonatal convulsions

| *Description* | *Trial* | *MgSO4* | *Control* | *RR* | *LCL* | *UCL* | *P: Hetero geneity†* |
| --- | --- | --- | --- | --- | --- | --- | --- |
| Neonatal convulsions (available data) | Crowther | 25/ 629 (3.97%) | 32/ 626 (5.11%) | 0.74 | 0.44 | 1.26 | . |
|  | Marret | 7/ 335 (2.09%) | 9/ 309 (2.91%) | 0.72 | 0.27 | 1.90 | . |
|  | Mittendorf | 1/ 71 (1.41%) | 4/ 75 (5.33%) | 0.26 | 0.03 | 2.31 | . |
|  | Magpie | 12/ 678 (1.77%) | 15/ 676 (2.22%) | 0.80 | 0.38 | 1.69 | . |
|  | Rouse | 23/1171 (1.96%) | 29/1244 (2.33%) | 0.79 | 0.45 | 1.38 | . |
|  | **OVERALL** | 68/2884 (2.36%) | 89/2930 (3.04%) | 0.75 | 0.54 | 1.03 | 0.9082 |

W=inverse variance; RR=Relative Risk; LCL = 95% Lower confidence limit; UCL = 95% Upper confidence limit; IPD=Individual patient data;

† Heterogeneity p values for one-stage analyses are from Wald chi-square tests for the interaction between treatment and trial in a GEE model.

Table 13 Intraventricular haemorrhage (any)

| *Description* | *Trial* | *MgSO4* | *Control* | *RR* | *LCL* | *UCL* | *P: Hetero geneity†* |
| --- | --- | --- | --- | --- | --- | --- | --- |
| Intraventricular haemorrhage (available data) | Crowther | 165/ 629 (26.2%) | 148/ 626 (23.6%) | 1.12 | 0.92 | 1.36 | . |
|  | Marret | 67/ 337 (19.9%) | 72/ 320 (22.5%) | 0.86 | 0.63 | 1.17 | . |
|  | Mittendorf | 11/ 71 (15.5%) | 10/ 72 (13.9%) | 1.12 | 0.50 | 2.47 | . |
|  | Rouse | 218/1112 (19.6%) | 252/1184 (21.3%) | 0.91 | 0.78 | 1.08 | . |
|  | **OVERALL** | 461/2149 (21.5%) | 482/2202 (21.9%) | 0.98 | 0.87 | 1.09 | 0.3718 |

W=inverse variance; RR=Relative Risk; LCL = 95% Lower confidence limit; UCL = 95% Upper confidence limit; IPD=Individual patient data; Overall two-stage RR estimates were calculated using a two-stage aggregate meta-analysis approach with inverse variance weighting.

† Heterogeneity p values for one-stage analyses are from Wald chi-square tests for the interaction between treatment and trial in a GEE model.

Table 14 Severe intraventricular haemorrhage (Grade 3 or 4)

| *Description* | *Trial* | *MgSO4* | *Control* | *RR* | *LCL* | *UCL* | *P: Hetero geneity†* |
| --- | --- | --- | --- | --- | --- | --- | --- |
| Severe IVH (grade 3 or 4)(available data) | Crowther | 50/ 629 (7.95%) | 50/ 626 (7.99%) | 0.97 | 0.66 | 1.44 | . |
|  | Marret | 22/ 337 (6.53%) | 23/ 320 (7.19%) | 0.85 | 0.47 | 1.53 | . |
|  | Mittendorf | 1/ 71 (1.41%) | 3/ 72 (4.17%) | 0.34 | 0.04 | 3.19 | . |
|  | Rouse | 23/1112 (2.07%) | 38/1184 (3.21%) | 0.64 | 0.38 | 1.06 | . |
|  | **OVERALL** | 96/2149 (4.47%) | 114/2202 (5.18%) | 0.83 | 0.63 | 1.09 | 0.4788 |

W=inverse variance; RR=Relative Risk; LCL = 95% Lower confidence limit; UCL = 95% Upper confidence limit; IPD=Individual patient data; † Heterogeneity p values for one-stage analyses are from Wald chi-square tests for the interaction between treatment and trial in a GEE model.

Table 15 Cystic periventricular leukomalacia

| *Description* | *Trial* | *MgSO4* | *Control* | *RR* | *LCL* | *UCL* | *P: Hetero geneity†* |
| --- | --- | --- | --- | --- | --- | --- | --- |
| Cystic periventricular leukomalacia (available data) | Crowther | 22/ 596 (3.69%) | 21/ 586 (3.58%) | 1.00 | 0.55 | 1.81 | . |
|  | Marret | 27/ 336 (8.04%) | 28/ 319 (8.78%) | 0.91 | 0.54 | 1.52 | . |
|  | Mittendorf | 1/64 | 0/65 |  |  |  |  |
|  | Rouse | 21/1112 (1.89%) | 27/1184 (2.28%) | 0.83 | 0.47 | 1.45 | . |
|  | **OVERALL** | 70/2044 (3.42%) | 76/2089 (3.64%) | 0.91 | 0.66 | 1.25 | 0.9025 |

W=inverse variance; RR=Relative Risk; LCL = 95% Lower confidence limit; UCL = 95% Upper confidence limit; IPD=Individual patient data;

† Heterogeneity p values for one-stage analyses are from Wald chi-square tests for the interaction between treatment and trial in a GEE model.

Table 16 Post haemorrhagic hydrocephaly or ventriculomegaly

| *Description* | *Trial* | *MgSO4* | *Control* | *RR* | *LCL* | *UCL* | *P: Hetero geneity†* |
| --- | --- | --- | --- | --- | --- | --- | --- |
| Post haemorrhagic hydrocephaly or vetriculomegaly (available data) | Crowther | 43/ 596 (7.21%) | 38/ 586 (6.48%) | 1.11 | 0.73 | 1.70 | . |
|  | Marret | 11/ 337 (3.26%) | 10/ 320 (3.13%) | 1.05 | 0.45 | 2.42 | . |
|  | Mittendorf | 3/ 67 (4.48%) | 5/ 70 (7.14%) | 0.76 | 0.18 | 3.27 | . |
|  | **OVERALL** | **57/1000 (5.70%)** | **53/976 (5.43%)** | **1.06** | **0.73** | **1.53** | **0.7770** |

W=inverse variance; RR=Relative Risk; LCL = 95% Lower confidence limit; UCL = 95% Upper confidence limit; IPD=Individual patient data;

† Heterogeneity p values for one-stage analyses are from Wald chi-square tests for the interaction between treatment and trial in a GEE model.

Table 17 Proven neonatal systemic infection

| *Description* | *Trial* | *MgSO4* | *Control* | *RR* | *LCL* | *UCL* | *P: Hetero geneity†* |
| --- | --- | --- | --- | --- | --- | --- | --- |
| Proven systemic infection (available data) | Crowther | 224/ 629 (35.6%) | 193/ 626 (30.8%) | 1.14 | 0.97 | 1.35 | . |
|  | Marret | 6/ 342 (1.75%) | 3/ 321 (0.93%) | 1.67 | 0.39 | 7.15 | . |
|  | Mittendorf | 2/ 71 (2.82%) | 1/ 75 (1.33%) | 2.11 | 0.20 | 22.86 | . |
|  | Rouse | 200/1171 (17.1%) | 220/1244 (17.7%) | 0.97 | 0.81 | 1.16 | . |
|  | **OVERALL** | 432/2213 (19.5%) | 417/2266 (18.4%) | 1.06 | 0.94 | 1.20 | 0.4654 |

W=inverse variance; RR=Relative Risk; LCL = 95% Lower confidence limit; UCL = 95% Upper confidence limit; IPD=Individual patient data;

† Heterogeneity p values for one-stage analyses are from Wald chi-square tests for the interaction between treatment and trial in a GEE model.

Table 18 Necrotising enterocolitis

| *Description* | *Trial* | *MgSO4* | *Control* | *RR* | *LCL* | *UCL* | *P: Hetero geneity†* |
| --- | --- | --- | --- | --- | --- | --- | --- |
| Necrotizing enterocolitis(available data) | Crowther | 30/ 629 (4.77%) | 31/ 626 (4.95%) | 0.96 | 0.59 | 1.57 | . |
|  | Marret | 9/ 342 (2.63%) | 6/ 320 (1.88%) | 1.41 | 0.51 | 3.90 | . |
|  | Mittendorf | 0/4 | 1/5 |  |  |  |  |
|  | Rouse | 116/1171 (9.91%) | 94/1244 (7.56%) | 1.27 | 0.97 | 1.66 | . |
|  | **OVERALL** | 155/2142 (7.24%) | 131/2190 (5.98%) | 1.22 | 0.97 | 1.53 | 0.5575 |

W=inverse variance; RR=Relative Risk; LCL = 95% Lower confidence limit; UCL = 95% Upper confidence limit; IPD=Individual patient data;

† Heterogeneity p values for one-stage analyses are from Wald chi-square tests for the interaction between treatment and trial in a GEE model.

Table 19 Patent ductus arteriosus requiring treatment

| *Description* | *Trial* | *MgSO4* | *Control* | *RR* | *LCL* | *UCL* | *P: Hetero geneity†* |
| --- | --- | --- | --- | --- | --- | --- | --- |
| Patent ductus arteriosus requiring treatment (available data) | Crowther | 224/ 629 (35.6%) | 216/ 626 (34.5%) | 1.07 | 0.91 | 1.25 | . |
|  | Marret | 40/ 342 (11.7%) | 37/ 319 (11.6%) | 0.89 | 0.55 | 1.42 | . |
|  | Mittendorf | 10/ 72 (13.9%) | 6/ 75 (8.00%) | 1.75 | 0.67 | 4.56 | . |
|  | Rouse | 151/1171 (12.9%) | 173/1244 (13.9%) | 0.95 | 0.77 | 1.18 | . |
|  | **OVERALL** | 425/2214 (19.2%) | 432/2264 (19.1%) | 1.03 | 0.91 | 1.16 | 0.4959 |

W=inverse variance; RR=Relative Risk; LCL = 95% Lower confidence limit; UCL = 95% Upper confidence limit; IPD=Individual patient data; † Heterogeneity p values for one-stage analyses are from Wald chi-square tests for the interaction between treatment and trial in a GEE model.

Table 20 Any retinopathy of prematurity

| *Description* | *Trial* | *MgSO4* | *Control* | *RR* | *LCL* | *UCL* | *P: Hetero geneity†* |
| --- | --- | --- | --- | --- | --- | --- | --- |
| Retinopathy of prematurity(available data) | Crowther | 188/ 524 (35.9%) | 172/ 508 (33.9%) | 1.08 | 0.90 | 1.29 | . |
|  | Marret | 12/ 271 (4.43%) | 9/ 262 (3.44%) | 1.47 | 0.58 | 3.72 | . |
|  | Mittendorf | 7/ 11 (63.6%) | 5/ 9 (55.6%) | 1.23 | 0.76 | 1.99 | . |
|  | Rouse | 263/1171 (22.5%) | 283/1244 (22.7%) | 0.98 | 0.84 | 1.15 | . |
|  | **OVERALL** | 470/1977 (23.8%) | 469/2023 (23.2%) | 1.03 | 0.92 | 1.15 | 0.7452 |

W=inverse variance; RR=Relative Risk; LCL = 95% Lower confidence limit; UCL = 95% Upper confidence limit; IPD=Individual patient data; † Heterogeneity p values for one-stage analyses are from Wald chi-square tests for the interaction between treatment and trial in a GEE model.

Table 21 Severe neonatal adverse outcome

| *Description* | *Trial* | *MgSO4* | *Control* | *RR* | *LCL* | *UCL* | *P: Hetero geneity†* |
| --- | --- | --- | --- | --- | --- | --- | --- |
| Severe neonatal adverse outcome(available data) | Crowther | 421/ 629 (66.9%) | 413/ 626 (66.0%) | 1.02 | 0.94 | 1.11 | . |
|  | Marret | 98/ 352 (27.8%) | 102/ 336 (30.4%) | 0.88 | 0.69 | 1.13 | . |
|  | Mittendorf | 17/ 72 (23.6%) | 14/ 76 (18.4%) | 1.31 | 0.69 | 2.50 | . |
|  | Rouse | 485/1172 (41.4%) | 482/1244 (38.7%) | 1.07 | 0.97 | 1.18 | . |
|  | **OVERALL** | 1021/2225 (45.9%) | 1011/2282 (44.3%) | 1.03 | 0.97 | 1.10 | 0.4411 |

W=inverse variance; RR=Relative Risk; LCL = 95% Lower confidence limit; UCL = 95% Upper confidence limit; IPD=Individual patient data; Severe neonatal adverse outcome (death, chronic lung disease, patent ductus arteriosus requiring treatment, neonatal encephalopathy, necrotising enterocolitis, stage 3 or worse retinopathy of prematurity, grade 3 or 4 IVH)

† Heterogeneity p values for one-stage analyses are from Wald chi-square tests for the interaction between treatment and trial in a GEE model.

Table 22 Developmental Delay or Intellectual Impairment at follow up

| *Description* | *Trial* | *MgSO4* | *Control* | *RR* | *LCL* | *UCL* | *P: Hetero geneity†* |
| --- | --- | --- | --- | --- | --- | --- | --- |
| Developmental delay (any)(available data) | Crowther | 176/ 498 (35.3%) | 173/ 483 (35.8%) | 1.00 | 0.84 | 1.19 | . |
|  | Marret | 57/ 312 (18.3%) | 62/ 291 (21.3%) | 0.88 | 0.62 | 1.24 | . |
|  | Magpie | 8/ 236 (3.39%) | 11/ 255 (4.31%) | 0.78 | 0.32 | 1.90 | . |
|  | Rouse | 433/ 939 (46.1%) | 460/ 998 (46.1%) | 1.01 | 0.91 | 1.11 | . |
|  | **OVERALL** | 674/1985 (34.0%) | 706/2027 (34.8%) | 0.99 | 0.91 | 1.08 | 0.8625 |

W=inverse variance; RR=Relative Risk; LCL = 95% Lower confidence limit; UCL = 95% Upper confidence limit; IPD=Individual patient data; Developmental Delay or Intellectual Impairment at follow up (categorised as nil, mild, moderate or severe by trialist)

† Heterogeneity p values for one-stage analyses are from Wald chi-square tests for the interaction between treatment and trial in a GEE model.

Table 23 At least Moderate or at Least Severe Developmental Delay

| *DESCRIPTION* | *Trial* | *MgSO4* | *Control* | *RR* | *LCL* | *UCL* | *P: Hetero geneity* |
| --- | --- | --- | --- | --- | --- | --- | --- |
| At least moderate developmental delay (available data) | Crowther | 79/ 497 (15.9%) | 67/ 479 (14.0%) | 1.18 | 0.86 | 1.62 | . |
|  | Marret | 7/ 312 (2.24%) | 4/ 291 (1.37%) | 1.64 | 0.48 | 5.54 | . |
|  | OVERALL | 86/809 (10.6%) | 71/770 (9.22%) | 1.20 | 0.88 | 1.63 | 0.5356 |
|  |  |  |  | . | . | . | . |
| At least severe developmental delay (available data) | Crowther | 32/ 497 (6.44%) | 33/ 479 (6.89%) | 0.97 | 0.59 | 1.57 | . |
|  | Marret | 1/ 312 (0.32%) | 1/ 291 (0.34%) | 0.93 | 0.06 | 14.87 | . |
|  | OVERALL | 33/809 (4.08%) | 34/770 (4.42%) | 0.96 | 0.59 | 1.55 | 0.9856 |
|  |  |  |  | . | . | . | . |

Table 24 Blindness

| *Description* | *Trial* | *MgSO4* | *Control* | *RR* | *LCL* | *UCL* | *P: Hetero geneity†* |
| --- | --- | --- | --- | --- | --- | --- | --- |
| Blindness(available data) | Crowther | 1/ 411 (0.24%) | 1/ 398 (0.25%) | 0.97 | 0.06 | 15.47 | . |
|  | Marret | 1/ 299 (0.33%) | 1/ 283 (0.35%) | 0.95 | 0.06 | 15.09 | . |
|  | Magpie | 1/ 236 (0.42%) | 2/ 255 (0.78%) | 0.54 | 0.05 | 5.92 | . |
|  | Rouse | 89/ 386 (23.1%) | 124/ 447 (27.7%) | 0.84 | 0.66 | 1.07 | . |
|  | **OVERALL** | 92/1332 (6.91%) | 128/1383 (9.26%) | 0.83 | 0.65 | 1.06 | 0.9870 |

W=inverse variance; RR=Relative Risk; LCL = 95% Lower confidence limit; UCL = 95% Upper confidence limit; IPD=Individual patient data;

† Heterogeneity p values for one-stage analyses are from Wald chi-square tests for the interaction between treatment and trial in a GEE model.

Table 25 Deafness

| *Description* | *Trial* | *MgSO4* | *Control* | *RR* | *LCL* | *UCL* | *P: Hetero geneity†* |
| --- | --- | --- | --- | --- | --- | --- | --- |
| Deafness(available data) | Crowther | 8/ 429 (1.86%) | 7/ 429 (1.63%) | 1.14 | 0.42 | 3.13 | . |
|  | Marret | 0/301 | 4/287 |  |  |  |  |
|  | Magpie | 1/ 236 (0.42%) | 1/ 255 (0.39%) | 1.08 | 0.07 | 17.18 | . |
|  | Mittendorf | 1/14 | 0/10 |  |  |  |  |
|  | Rouse | 74/ 429 (17.2%) | 75/ 483 (15.5%) | 1.07 | 0.80 | 1.45 | . |
|  | **OVERALL** | 83/1094 (7.59%) | 83/1167 (7.11%) | 1.10 | 0.83 | 1.47 | 0.9899 |

W=inverse variance; RR=Relative Risk; LCL = 95% Lower confidence limit; UCL = 95% Upper confidence limit; IPD=Individual patient data;

† Heterogeneity p values for one-stage analyses are from Wald chi-square tests for the interaction between treatment and trial in a GEE model.

Table 26 Gross motor dysfunction (any)

| *Description* | *Trial* | *MgSO4* | *Control* | *RR* | *LCL* | *UCL* | *P: Hetero geneity†* |
| --- | --- | --- | --- | --- | --- | --- | --- |
| Gross motor dysfunction delay (any) (available data) | Crowther | 102/ 529 (19.3%) | 107/ 513 (20.9%) | 0.92 | 0.71 | 1.18 | . |
|  | Marret | 66/ 313 (21.1%) | 70/ 292 (24.0%) | 0.89 | 0.65 | 1.22 | . |
|  | Rouse | 71/ 865 (8.21%) | 102/ 910 (11.2%) | 0.73 | 0.55 | 0.98 | . |
|  | **OVERALL** | 239/1707 (14.0%) | 279/1715 (16.3%) | 0.85 | 0.72 | 1.00 | 0.4731 |

W=inverse variance; RR=Relative Risk; LCL = 95% Lower confidence limit; UCL = 95% Upper confidence limit; IPD=Individual patient data; Gross motor dysfunction (any) (defined as mild, moderate or severe, by trialists or by the Gross Motor Classification System [score 1-5], if available) † Heterogeneity p values for one-stage analyses are from Wald chi-square tests for the interaction between treatment and trial in a GEE model.

Table 27 Gross motor dysfunction (moderate or severe)

| *Description* | *Trial* | *MgSO4* | *Control* | *RR* | *LCL* | *UCL* | *P: Hetero geneity†* |
| --- | --- | --- | --- | --- | --- | --- | --- |
| Gross motor dysfunction delay (mod/sev) (available data) | Crowther | 11/ 529 (2.08%) | 10/ 513 (1.95%) | 1.07 | 0.46 | 2.49 | . |
|  | Marret | 19/ 313 (6.07%) | 22/ 292 (7.53%) | 0.80 | 0.44 | 1.48 | . |
|  | Rouse | 25/ 865 (2.89%) | 38/ 910 (4.18%) | 0.69 | 0.42 | 1.14 | . |
|  | **OVERALL** | 55/1707 (3.22%) | 70/1715 (4.08%) | 0.78 | 0.55 | 1.11 | 0.6855 |

W=inverse variance; RR=Relative Risk; LCL = 95% Lower confidence limit; UCL = 95% Upper confidence limit; IPD=Individual patient data;

† Heterogeneity p values for one-stage analyses are from Wald chi-square tests for the interaction between treatment and trial in a GEE model.

Table 28 Neurosensory disability

| *Description* | *Trial* | *MgSO4* | *Control* | *RR* | *LCL* | *UCL* | *P: Hetero geneity†* |
| --- | --- | --- | --- | --- | --- | --- | --- |
| Neurosensory disability (available data) | Crowther | 195/ 533 (36.6%) | 193/ 514 (37.5%) | 1.00 | 0.85 | 1.17 | . |
|  | Marret | 69/ 313 (22.0%) | 82/ 293 (28.0%) | 0.79 | 0.59 | 1.07 | . |
|  | Mittendorf | 4/ 61 (6.56%) | 3/ 62 (4.84%) | 1.35 | 0.31 | 5.83 | . |
|  | Magpie | 8/ 236 (3.39%) | 11/ 255 (4.31%) | 0.79 | 0.32 | 1.92 | . |
|  | Rouse | 519/1140 (45.5%) | 562/1215 (46.3%) | 0.99 | 0.90 | 1.08 | . |
|  | **OVERALL** | 795/2283 (34.8%) | 851/2339 (36.4%) | 0.98 | 0.90 | 1.05 | 0.6500 |

W=inverse variance; RR=Relative Risk; LCL = 95% Lower confidence limit; UCL = 95% Upper confidence limit; IPD=Individual patient data; Neurosensory disability (any developmental delay or intellectual impairment, cerebral palsy, blindness, or deafness)

† Heterogeneity p values for one-stage analyses are from Wald chi-square tests for the interaction between treatment and trial in a GEE model.

Table 29 Major neurosensory disability

| *Description* | *Trial* | *MgSO4* | *Control* | *RR* | *LCL* | *UCL* | *P: Hetero geneity†* |
| --- | --- | --- | --- | --- | --- | --- | --- |
| Major neurosensory disability(available data) | Crowther | 91/ 533 (17.1%) | 80/ 514 (15.6%) | 1.16 | 0.87 | 1.54 | . |
|  | Marret | 14/ 313 (4.47%) | 21/ 293 (7.17%) | 0.63 | 0.32 | 1.21 | . |
|  | Magpie | 8/ 236 (3.39%) | 11/ 255 (4.31%) | 0.79 | 0.32 | 1.92 | . |
|  | Rouse | 291/1140 (25.5%) | 315/1215 (25.9%) | 0.99 | 0.86 | 1.14 | . |
|  | **OVERALL** | 404/2222 (18.2%) | 427/2277 (18.8%) | 1.00 | 0.88 | 1.13 | 0.4301 |

W=inverse variance; RR=Relative Risk; LCL = 95% Lower confidence limit; UCL = 95% Upper confidence limit; IPD=Individual patient data; Major neurosensory disability (developmental delay or intellectual impairment level 2 or 3, cerebral palsy moderate or severe, blindness, or deafness)

† Heterogeneity p values for one-stage analyses are from Wald chi-square tests for the interaction between treatment and trial in a GEE model.

Table 30 Psychomotor dysfunction

| *Description* | *Trial* | *MgSO4* | *Control* | *RR* | *LCL* | *UCL* | *P: Hetero geneity†* |
| --- | --- | --- | --- | --- | --- | --- | --- |
| Psychomotor dysfunction (available data) | Crowther | 179/ 482 (37.1%) | 162/ 461 (35.1%) | 1.07 | 0.89 | 1.28 | . |
|  | Rouse | 313/ 939 (33.3%) | 335/ 998 (33.6%) | 1.01 | 0.88 | 1.14 | . |
|  | **OVERALL** | 492/1421 (34.6%) | 497/1459 (34.1%) | 1.03 | 0.92 | 1.14 | 0.6085 |

W=inverse variance; RR=Relative Risk; LCL = 95% Lower confidence limit; UCL = 95% Upper confidence limit; IPD=Individual patient data; † Heterogeneity p values for one-stage analyses are from Wald chi-square tests for the interaction between treatment and trial in a GEE model. Psychomotor dysfunction (categorised as nil, mild (less than 85), moderate (less than 70) or severe (less than 55) by the Psychomotor Development Index (PDI) on the Bayley Scales of Infant Development)

Table 31 Death or major neurosensory disability

| *Description* | *Trial* | *MgSO4* | *Control* | *RR* | *LCL* | *UCL* | *P: Hetero geneity†* |
| --- | --- | --- | --- | --- | --- | --- | --- |
| Death or major neurosensory disability(available data) | Crowther | 178/ 629 (28.3%) | 188/ 626 (30.0%) | 0.97 | 0.81 | 1.16 | . |
|  | Marret | 48/ 353 (13.6%) | 59/ 338 (17.5%) | 0.77 | 0.54 | 1.12 | . |
|  | Mittendorf | 12/ 86 (14.0%) | 2/ 80 (2.50%) | 5.11 | 1.17 | 22.36 | . |
|  | Magpie | 208/ 790 (26.3%) | 188/ 785 (23.9%) | 1.07 | 0.90 | 1.27 | . |
|  | Rouse | 393/1188 (33.1%) | 411/1256 (32.7%) | 1.02 | 0.90 | 1.14 | . |
|  | **OVERALL** | 839/3046 (27.5%) | 848/3085 (27.5%) | 1.01 | 0.93 | 1.10 | 0.1277 |

W=inverse variance; RR=Relative Risk; LCL = 95% Lower confidence limit; UCL = 95% Upper confidence limit; IPD=Individual patient data; Death or major neurosensory disability (defined as any moderate or severe neurosensory impairment)

† Heterogeneity p values for one-stage analyses are from Wald chi-square tests for the interaction between treatment and trial in a GEE model.

Table 32 Death or any neurosensory disability

| *Description* | *Trial* | *MgSO4* | *Control* | *RR* | *LCL* | *UCL* | *P: Hetero geneity†* |
| --- | --- | --- | --- | --- | --- | --- | --- |
| Death or neurosensory disability (available data) | Crowther | 282/ 629 (44.8%) | 301/ 626 (48.1%) | 0.95 | 0.84 | 1.07 | . |
|  | Marret | 103/ 353 (29.2%) | 120/ 338 (35.5%) | 0.81 | 0.65 | 1.03 | . |
|  | Mittendorf | 14/ 86 (16.3%) | 4/ 80 (5.00%) | 3.06 | 1.04 | 8.99 | . |
|  | Magpie | 208/ 790 (26.3%) | 188/ 785 (23.9%) | 1.07 | 0.90 | 1.27 | . |
|  | Rouse | 621/1188 (52.3%) | 658/1256 (52.4%) | 1.00 | 0.93 | 1.09 | . |
|  | **OVERALL** | 1228/3046 (40.3%) | 1271/3085 (41.2%) | 0.99 | 0.93 | 1.05 | 0.0916 |

W=inverse variance; RR=Relative Risk; LCL = 95% Lower confidence limit; UCL = 95% Upper confidence limit; IPD=Individual patient data; Death or any neurosensory disability (any developmental delay or intellectual impairment, cerebral palsy, blindness, or deafness)

† Heterogeneity p values for one-stage analyses are from Wald chi-square tests for the interaction between treatment and trial in a GEE model.

Table 33 Death or moderate-severe gross motor dysfunction

| *Description* | *Trial* | *MgSO4* | *Control* | *RR* | *LCL* | *UCL* | *P: Hetero geneity†* |
| --- | --- | --- | --- | --- | --- | --- | --- |
| Death or mod-severe gross motor dysfunction (palisano) (available data) | Crowther | 98/ 629 (15.6%) | 118/ 626 (18.8%) | 0.83 | 0.65 | 1.08 | . |
|  | Marret | 53/ 353 (15.0%) | 60/ 338 (17.8%) | 0.82 | 0.57 | 1.17 | . |
|  | Rouse | 130/1188 (10.9%) | 134/1256 (10.7%) | 1.03 | 0.81 | 1.30 | . |
|  | **OVERALL** | 281/2170 (12.9%) | 312/2220 (14.1%) | 0.91 | 0.78 | 1.06 | 0.4048 |

W=inverse variance; RR=Relative Risk; LCL = 95% Lower confidence limit; UCL = 95% Upper confidence limit; IPD=Individual patient data; † Heterogeneity p values for one-stage analyses are from Wald chi-square tests for the interaction between treatment and trial in a GEE model. Death or moderate-severe gross motor dysfunction (such that the child was not walking at age two years or later, or the inability to grasp and release a small block with both hands at two years or later)

Table 34 Length of maternal postnatal stay

|  | *MgSO4* | | *Control* | | |  | | | | | | |
| --- | --- | --- | --- | --- | --- | --- | --- | --- | --- | --- | --- | --- |
| *TRIAL* | *n* | *Geometric Mean Days(95% CI)* | | *n* | *Geometric Mean Days (95% CI)* | | *Relative Difference* | *LCL* | *UCL* | *P VALUE* | *P: HETERO GENEITY* |  |
| Crowther | 535 | 4.34 (4.12, 4.57) | | 527 | 4.32 (4.12, 4.54) | | 1.00 | 0.94 | 1.08 |  | . |  |
| Mittendorf | 72 | 2.03 (1.75, 2.36) | | 69 | 2.00 (1.76, 2.28) | | 1.02 | 0.84 | 1.23 |  | . |  |
| Magpie | 745 | 8.17 (7.76, 8.60) | | 741 | 8.14 (7.74, 8.55) | | 1.00 | 0.93 | 1.08 |  | . |  |
| Rouse | 1086 | 10.29 (9.75, 10.86) | | 1141 | 10.09 (9.56, 10.65) | | 1.02 | 0.95 | 1.10 |  | . |  |
| **OVERALL** | 2438 | 7.56 (7.30, 7.83) | | 2478 | 7.55 (7.30, 7.82) | | 1.01 | 0.97 | 1.06 | 0.6121 | 0.9889 |  |

Data for length of stay were highly skewed therefore log transformed prior to analysis

Mothers recorded as 0 days in hospital were changed to 1 day in hospital prior to analysis

The relative difference can be interpreted in the same way as a relative risk and represents the proportional increase or decrease due to treatment. The relative difference is calculated by exponentiating the difference between the group means calculated on the log scale.

Table 35 Gestational age at birth (weeks)

|  | | *MgSO4* | | *Control* | |  | | | | |
| --- | --- | --- | --- | --- | --- | --- | --- | --- | --- | --- |
| *DESCRIPTION* | *TRIAL* | *n* | *Mean Weeks*  *(95% CI)* | *n* | *Mean Weeks*  *(95% CI)* | *Mean Diff* | *LCL* | *UCL* | *P VALUE* | *P: HET* |
| Gest age at birth | Crowther | 629 | 27.10 (26.93, 27.26) | 626 | 27.04 (26.87, 27.21) | 0.06 | -0.18 | 0.29 | 0.6497 | . |
|  | Marret | 352 | 29.38 (29.15, 29.61) | 336 | 29.46 (29.22, 29.69) | -0.08 | -0.41 | 0.25 | 0.6296 | . |
|  | Mittendorf | 54 | 32.43 (31.52, 33.33) | 60 | 32.15 (31.29, 33.01) | 0.28 | -0.94 | 1.49 | 0.6560 | . |
|  | Magpie | 782 | 33.29 (33.04, 33.54) | 783 | 33.36 (33.11, 33.61) | -0.07 | -0.42 | 0.28 | 0.7035 | . |
|  | Rouse | 1179 | 29.37 (29.19, 29.55) | 1252 | 29.28 (29.11, 29.45) | 0.09 | -0.16 | 0.33 | 0.4922 | . |
|  | **OVERALL** | 2996 | 29.97 (29.84, 30.10) | 3057 | 29.94 (29.81, 30.07) | 0.02 | -0.13 | 0.17 | 0.7499 | 0.9540 |

Table 36 Growth measurements at birth

|  | | ***MgSO4*** | | ***Control*** | |  | | | | |
| --- | --- | --- | --- | --- | --- | --- | --- | --- | --- | --- |
| ***Description*** | ***TRIAL*** | ***n*** | ***Mean*^b^ *(SE)*** | ***n*** | ***Mean*^b^ *(SE)*** | ***Mean^c^ Diff*** | ***LCL*** | ***UCL*** | ***P VALUE*** | ***P: HETERO GENEITY*** |
| Birth weight z score | Crowther | 628 | -0.10 (0.04) | 626 | -0.08 (0.04) | -0.03 | -0.14 | 0.09 | . | . |
|  | Marret | 351 | -0.02 (0.04) | 334 | 0.04 (0.04) | -0.04 | -0.16 | 0.08 | . | . |
|  | Mittendorf | 54 | -0.63 (0.10) | 60 | -0.65 (0.12) | 0.01 | -0.30 | 0.33 | . | . |
|  | Magpie | 777 | -0.79 (0.04) | 775 | -0.76 (0.04) | -0.02 | -0.14 | 0.09 | . | . |
|  | Rouse | 1177 | -0.02 (0.02) | 1252 | 0.07 (0.02) | -0.09 | -0.15 | -0.02 | . | . |
|  | **OVERALL** | 2987 | -0.25 (0.02) | 3047 | -0.19 (0.02) | -0.05 | -0.10 | -0.00 | 0.0399 | 0.8207 |
|  |  | . |  | . |  | . | . | . | . | . |
| Birth weight ^a^ (exclusions as per z score anal) | Crowther | 628 | 1021.09 (14.77) | 626 | 1019.38 (14.79) | -1.94 | -46.62 | 42.75 | . | . |
|  | Marret | 351 | 1372.48 (20.35) | 334 | 1405.81 (21.42) | -21.10 | -85.08 | 42.88 | . | . |
|  | Mittendorf | 54 | 1763.26 (85.27) | 60 | 1702.45 (81.91) | 58.88 | -182.1 | 299.90 | . | . |
|  | Magpie | 777 | 1905.75 (27.90) | 775 | 1927.26 (27.38) | -18.74 | -97.40 | 59.93 | . | . |
|  | Rouse | 1177 | 1410.70 (16.58) | 1252 | 1420.88 (16.32) | -6.82 | -54.73 | 41.08 | . | . |
|  | **OVERALL** | 2987 | 1459.44 (12.04) | 3047 | 1471.08 (11.94) | -9.69 | -40.76 | 21.37 | 0.5408 | 0.9566 |
|  |  | . |  | . |  | . | . | . | . | . |
| Head circumference | Crowther | 501 | 25.27 (0.11) | 487 | 25.16 (0.12) | 0.09 | -0.26 | 0.44 | . | . |
|  | Marret | 317 | 27.51 (0.14) | 306 | 27.49 (0.15) | 0.09 | -0.35 | 0.52 | . | . |
|  | Mittendorf | 71 | 29.04 (0.40) | 72 | 29.37 (0.42) | -0.35 | -1.54 | 0.84 | . | . |
|  | Rouse | 1149 | 27.04 (0.09) | 1226 | 27.07 (0.09) | -0.01 | -0.27 | 0.26 | . | . |
|  | **OVERALL** | 2038 | 26.75 (0.07) | 2091 | 26.77 (0.07) | 0.02 | -0.17 | 0.21 | 0.8529 | 0.8703 |
|  |  | . |  | . |  | . | . | . | . | . |
| Length | Crowther | 459 | 35.34 (0.18) | 442 | 35.46 (0.20) | -0.24 | -0.79 | 0.32 | . | . |
|  | Marret | 264 | 39.33 (0.19) | 257 | 39.57 (0.21) | -0.19 | -0.79 | 0.42 | . | . |
|  | Mittendorf | 69 | 42.50 (0.59) | 71 | 42.53 (0.74) | 0.05 | -1.90 | 1.99 | . | . |
|  | Rouse | 1162 | 39.10 (0.14) | 1239 | 39.09 (0.14) | 0.06 | -0.34 | 0.46 | . | . |
|  | **OVERALL** | 1954 | 38.37 (0.11) | 2009 | 38.47 (0.11) | -0.03 | -0.33 | 0.26 | 0.8187 | 0.8522 |

SE = standard error

a Slightly skewed distribution.

b Unadjusted (raw) means

c Adjusted mean difference ( MgSO4-Control)

Table 37 Growth measurements at follow up

|  | | *MgSO4* | | *Control* | |  | | | | |
| --- | --- | --- | --- | --- | --- | --- | --- | --- | --- | --- |
| *Description* | *TRIAL* | *n* | *Mean***^a^** *(SE)* | *n* | *Mean***^a^** *(SE)* | *Mean Diff***^b^** | *LCL* | *UCL* | *P VALUE* | *P: HETERO GENEITY* |
| Weight (adjusted for age and sex) | Crowther | 521 | 12.12 (0.08) | 503 | 12.09 (0.08) | 0.01 | -0.21 | 0.23 | . | . |
|  | Marret | 302 | 11.64 (0.09) | 278 | 11.53 (0.10) | 0.12 | -0.16 | 0.39 | . | . |
|  | **OVERALL** | **823** | **11.94 (0.06)** | **781** | **11.89 (0.06)** | **0.05** | **-0.13** | **0.22** | **0.6014** | **0.5306** |
|  |  | . |  | . |  | . | . | . | . | . |
| Head circ (adjusted for age and sex) | Crowther | 507 | 48.62 (0.09) | 484 | 48.60 (0.09) | 0.04 | -0.20 | 0.28 | . | . |
|  | Marret | 272 | 48.59 (0.10) | 248 | 48.32 (0.10) | 0.26 | -0.02 | 0.55 | . | . |
|  | **OVERALL** | **779** | **48.61 (0.07)** | **732** | **48.51 (0.07)** | **0.11** | **-0.07** | **0.30** | **0.2355** | **0.2387** |
|  |  | . |  | . |  | . | . | . | . | . |
| Height (adjusted for age and sex) | Crowther | 409 | 86.49 (0.24) | 379 | 86.39 (0.25) | -0.03 | -0.64 | 0.58 | . | . |
|  | Marret | 299 | 85.21 (0.27) | 275 | 85.68 (0.29) | -0.49 | -1.30 | 0.32 | . | . |
|  | **OVERALL** | **708** | **85.95 (0.18)** | **654** | **86.09 (0.19)** | **-0.22** | **-0.71** | **0.27** | **0.3781** | **0.3836** |
|  |  | . |  | . |  | . | . | . | . | . |
| Follow up weight z score | Crowther | 521 | -0.40 (0.05) | 503 | -0.43 (0.05) | 0.01 | -0.14 | 0.16 | . | . |
|  | Marret | 302 | -0.21 (0.07) | 277 | -0.29 (0.07) | 0.08 | -0.12 | 0.29 | . | . |
|  | **OVERALL** | **823** | **-0.33 (0.04)** | **780** | **-0.38 (0.04)** | **0.04** | **-0.09** | **0.16** | **0.5723** | **0.5503** |
|  |  | . |  | . |  | . | . | . | . | . |
| Follow up Length/Height z score | Crowther | 409 | -0.96 (0.06) | 379 | -0.96 (0.06) | 0.00 | -0.18 | 0.19 | . | . |
|  | Marret | 299 | -0.38 (0.08) | 274 | -0.24 (0.09) | -0.15 | -0.42 | 0.11 | . | . |
|  | **OVERALL** | **708** | **-0.72 (0.05)** | **653** | **-0.66 (0.06)** | **-0.06** | **-0.21** | **0.09** | **0.4410** | **0.3369** |
|  |  | . |  | . |  | . | . | . | . | . |

a Unadjusted (raw) means

b Adjusted mean difference ( MgSO4-Control)

Table 38 Adverse event enough to stop treatment

| *Description* | *Trial* | *MgSO4* | *Control* | *RR* | *LCL* | *UCL* | *P: Hetero geneity†* |
| --- | --- | --- | --- | --- | --- | --- | --- |
| Adverse event stop treatment | Crowther | 21/ 522 (4.02%) | 4/ 509 (0.79%) | 5.12 | 1.77 | 14.81 | . |
|  | Marret | 0/ 268 (0.00%) | 0/ 259 (0.00%) | . | . | . | . |
|  | Mittendorf | 0/ 63 (0.00%) | 0/ 31 (0.00%) | . | . | . | . |
|  | Magpie | 87/ 710 (12.3%) | 50/ 703 (7.11%) | 1.72 | 1.24 | 2.40 | . |
|  | Rouse | 7/ 1078 (0.65%) | 4/ 1125 (0.36%) | 1.83 | 0.54 | 6.22 | . |
|  | **OVERALL** | 115/2310 (4.98%) | 58/2337 (2.48%) | 1.95 | 1.44 | 2.65 | 0.1081 |

Table 39 Intrapartum fever requiring antibiotics

| *Description* | *Trial* | *MgSO4* | *Control* | *RR* | *LCL* | *UCL* | *P: Hetero geneity†* |
| --- | --- | --- | --- | --- | --- | --- | --- |
| Intrapartum fever requiring | Crowther | 179/ 535 (33.5%) | 177/ 527 (33.6%) | 1.00 | 0.84 | 1.18 | . |
|  | Mittendorf | 58/ 68 (85.3%) | 50/ 65 (76.9%) | 1.11 | 0.94 | 1.31 | . |
|  | **OVERALL** | **237/603 (39.3%)** | **227/592 (38.3%)** | **1.05** | **0.94** | **1.18** | **0.3733** |

Table 40 Postpartum haemorrhage (>=500ml)

| *Description* | *Trial* | *MgSO4* | *Control* | *RR* | *LCL* | *UCL* | *P: Hetero geneity†* |
| --- | --- | --- | --- | --- | --- | --- | --- |
| Postpartum haemorrhage (>=500ml) | Crowther | 156/ 535 (29.2%) | 151/ 527 (28.7%) | 1.02 | 0.84 | 1.23 | . |
|  | Marret | 1/ 290 (0.34%) | 1/ 283 (0.35%) | 0.98 | 0.06 | 15.53 | . |
|  | Mittendorf | 0/ 69 (0.00%) | 0/ 65 (0.00%) | . | . | . | . |
|  | Magpie | 253/ 632 (40.0%) | 258/ 648 (39.8%) | 1.01 | 0.88 | 1.15 | . |
|  | **OVERALL** | **410/1457 (28.1%)** | **410/1458 (28.1%)** | **1.01** | **0.90** | **1.13** | **0.9945** |

Table 41 Mode of Birth (Caesarean)

| *Description* | *Trial* | *MgSO4* | *Control* | *RR* | *LCL* | *UCL* | *P: Hetero geneity†* |
| --- | --- | --- | --- | --- | --- | --- | --- |
| Mode of birth (Caesarean) | Crowther | 287/ 535 (53.6%) | 289/ 527 (54.8%) | 0.98 | 0.88 | 1.09 | . |
|  | Marret | 116/ 286 (40.6%) | 96/ 278 (34.5%) | 1.17 | 0.95 | 1.46 | . |
|  | Mittendorf | 19/ 74 (25.7%) | 18/ 70 (25.7%) | 1.00 | 0.57 | 1.74 | . |
|  | Magpie | 483/ 746 (64.7%) | 449/ 750 (59.9%) | 1.08 | 1.00 | 1.17 | . |
|  | Rouse | 405/ 1086 (37.3%) | 430/ 1141 (37.7%) | 0.99 | 0.89 | 1.10 | . |
|  | **OVERALL** | 1310/2727 (48.0%) | 1282/2766 (46.3%) | 1.04 | 0.98 | 1.10 | 0.3777 |

Table 42 Chorioamnionitis during labour

| *Description* | *Trial* | *MgSO4* | *Control* | *RR* | *LCL* | *UCL* | *P: Hetero geneity†* |
| --- | --- | --- | --- | --- | --- | --- | --- |
| Chorioamnionitis | Crowther | 179/ 535 (33.5%) | 177/ 527 (33.6%) | 1.00 | 0.84 | 1.18 | . |
|  | Mittendorf | 17/ 74 (23.0%) | 18/ 70 (25.7%) | 0.89 | 0.50 | 1.59 | . |
|  | Rouse | 127/ 1086 (11.7%) | 131/ 1141 (11.5%) | 1.02 | 0.81 | 1.28 | . |
|  | **OVERALL** | 323/1695 (19.1%) | 326/1738 (18.8%) | 1.00 | 0.87 | 1.14 | 0.9174 |
