# Supplementary material for: Assessing the neuroprotective benefits for babies of antenatal magnesium sulphate: An individual participant data meta-analysis
Source: PLoS Med. 2017 Oct 4;14(10):e1002398. doi: 10.1371/journal.pmed.1002398 (PMC5627896; doi:10.1371/journal.pmed.1002398)
Supplement: S3 Text — (DOCX) [file pmed.1002398.s008.docx]

**S3 Text, Data items collected**

Data items collected were agreed by the AMICABLE Group.

**Trial level information**

1. Dates the trial opened and closed accrual

2. Number of women randomised

3. Informed consent procedures

4. Methods of random allocation

5. Stratification factors used

6. Methods of allocation concealment

7. Blinding of outcome assessment

8. Purpose magnesium sulphate treatment given (neuroprotection for the fetus, neuroprotection for the mother, tocolysis, pre-eclampsia, other)

9. Details of the planned intervention in the experimental arm

10. Details of the planned intervention in the control arm

**Participant-level information: maternal characteristics at trial entry**

1. Unique identification coded for anonymity

2. Maternal age

3. Parity

4. Ethnicity

5. Public or private patient

6. Previous obstetric history

7. Reason the woman was considered to be at risk of preterm birth (such as preterm labour/pre-eclampsia/placenta abruption/placenta previa/chorioamnionitis/other antepartum haemorrhage/preterm rupture of membranes)

8. Number of fetuses in-utero (singleton, twin or higher order multiple pregnancy)

9. Gestational age at trial entry

**Participant-level information: maternal outcomes after trial entry**

1. Treatment dose details (loading dose given, maintenance dose, retreatment dose)

2. Time from antenatal magnesium sulphate treatment to birth

3. Adverse events for the woman at time of treatment

4. Intrapartum information

5. Postnatal information

**Participant-level information: infant outcomes**

1. Unique baby identification and mother identification coded for anonymity

2. Date and time of birth

3. Gestational age at birth

4. Sex

5. Mode of birth

6. Birth weight, length, head circumference

7. Apgar scores at 1 and 5 minutes

8. Neonatal complications/status

9. Mortality and age at death

10. Cause of death

11. Childhood follow-up assessments.
