# Supplementary material for: Assessing the neuroprotective benefits for babies of antenatal magnesium sulphate: An individual participant data meta-analysis
Source: PLoS Med. 2017 Oct 4;14(10):e1002398. doi: 10.1371/journal.pmed.1002398 (PMC5627896; doi:10.1371/journal.pmed.1002398)
Supplement: S4 Text — (DOCX) [file pmed.1002398.s009.docx]

**S4 Text, Pre-specified secondary outcomes**

Pre-specified secondary outcomes for the infant included: gestational age at birth, birth weight (raw values and Z scores) [1] head circumference at birth (raw values and Z scores) [1], length at birth (raw values and Z scores) [1], Apgar score <7 at five minutes, use of active resuscitation at birth, use of ongoing respiratory support, chronic lung disease/bronchopulmonary dysplasia (as defined by trialists), neonatal convulsions, neonatal encephalopathy, any intraventricular haemorrhage (IVH), severe IVH (grade 3 or 4), cystic periventricular leucomalacia, posthaemorrhagic hydrocephalus or ventriculomegaly, proven systemic neonatal infection, necrotising enterocolitis, patent ductus arteriosus requiring treatment and retinopathy of prematurity.

Childhood secondary outcomes included: death (fetal, neonatal or later death up to the time of follow up) and cause of death, cerebral palsy (any cerebral palsy, as defined by the trialists), severity of cerebral palsy (categorised as, mild, moderate or severe, as defined by the trialists), severe adverse neonatal outcome (defined as death, chronic lung disease, patent ductus arteriosus requiring treatment, neonatal encephalopathy, necrotising enterocolitis, stage 3 or worse retinopathy of prematurity, grade 3 or 4 IVH), other neurosensory impairments (other than cerebral palsy) including developmental delay or intellectual impairment (categorised as nil, mild, moderate or severe, by the trialists) blindness (defined as visual acuity worse than 6/60 (20/200) in the better eye), deafness (defined as hearing loss requiring amplification or worse), gross motor dysfunction (defined as mild, moderate or severe, by trialists or by the Gross Motor Classification System [score 1-5], if available) [2], psychomotor dysfunction (categorised as nil, mild (<85), moderate (<70) or severe (<55) by the Psychomotor Developmental Index (PDI)) on the Bayley Scales of Infant Development [3], any neurosensory disability (defined as developmental delay or intellectual impairment [developmental quotient or intelligence quotient more than one SD below the mean]; cerebral palsy [abnormality of muscle tone with motor dysfunction]; blindness; or deafness; at follow up later in childhood), major neurosensory disability (defined as any moderate or severe neurosensory impairment), death or substantial gross motor dysfunction (defined as death, or motor dysfunction (such that the child was not walking at age two years or later, or the inability to grasp and release a small block with both hands at two years or later)), death or neurosensory disability (defined as death or any of the neurosensory impairments), death or major neurosensory disability (defined as death or any moderate or severe neurosensory impairment) and growth assessments at childhood follow up for: weight (raw values and Z scores) [4], head circumference (raw values and Z scores) [4], height (raw values and Z scores) [4], and other developmental assessments (as used by the trialists).

For the women secondary outcomes included: adverse effects severe enough to stop treatment, postpartum haemorrhage, mode of birth, chorioamnionitis during labour, intrapartum fever requiring the use of antibiotics, length of postnatal stay and individual components of maternal primary outcome.

References

1. Roberts CL, Lancaster PAL: Australian national birthweight percentiles by gestational age. Med J Australia 1999, 170(3):114-118.

2. Palisano R, Rosenbaum P, Walter S, Russell D, Wood E, Galuppi B. Development and reliability of a system to classify gross motor function in children with cerebral palsy. Dev Med Child Neurol 1997, 39(4):214-223.

3. Bayley scales of infant development. 2nd edition. San Antonio: Psychological Corportation; 1993.

4. WHO Multicentre Growth Reference Study Group: WHO Child Growth Standards based on length/height, weight and age. Acta Paediatr 2006, 450:76-85.
